# Loss of Key EMT-Regulating miRNAs Highlight the Role of ZEB1 in EGFR Tyrosine Kinase Inhibitor-Resistant NSCLC

**DOI:** 10.3390/ijms241914742

**Published:** 2023-09-29

**Authors:** Linus Gohlke, Ahmad Alahdab, Angela Oberhofer, Karolina Worf, Stefan Holdenrieder, Martin Michaelis, Jindrich Cinatl, Christoph A Ritter

**Affiliations:** 1 Institute of Pharmacy, Clinical Pharmacy, University Greifswald, Friedrich-Ludwig-Jahn-Str. 17, 17489 Greifswald, Germany; ahmad.alahdab@uni-greifswald.de; 2 Munich Biomarker Research Center, Institute of Laboratory Medicine, German Heart Center, Technical University Munich, 80636 Munich, Germany; oberhofer@dhm.mhn.de (A.O.); karolina.worf@tum.de (K.W.); holdenrieder@dhm.mhn.de (S.H.); 3 School of Biosciences, Division of Natural Sciences, University of Kent, Canterbury, Kent CT2 7NJ, UK; m.michaelis@kent.ac.uk; 4 Institute of Medical Virology, University Hospital Frankfurt, Goethe University, 60596 Frankfurt am Main, Germany; cinatl@em.uni-frankfurt.de

**Keywords:** NSCLC, EMT, EMP-hybrid, miRNA, drug resistance, EGFR-mutated, EGFR inhibitor, miR-205-5p, ZEB1, extracellular vesicles, cancer stem cells

## Abstract

Despite recent advances in the treatment of non-small cell lung cancer (NSCLC), acquired drug resistance to targeted therapy remains a major obstacle. Epithelial-mesenchymal transition (EMT) has been identified as a key resistance mechanism in NSCLC. Here, we investigated the mechanistic role of key EMT-regulating small non-coding microRNAs (miRNAs) in sublines of the NSCLC cell line HCC4006 adapted to afatinib, erlotinib, gefitinib, or osimertinib. The most differentially expressed miRNAs derived from extracellular vesicles were associated with EMT, and their predicted target *ZEB1* was significantly overexpressed in all resistant cell lines. Transfection of a miR-205-5p mimic partially reversed EMT by inhibiting *ZEB1*, restoring *CDH1* expression, and inhibiting migration in erlotinib-resistant cells. Gene expression of EMT-markers, transcription factors, and miRNAs were correlated during stepwise osimertinib adaptation of HCC4006 cells. Temporally relieving cells of osimertinib reversed transition trends, suggesting that the implementation of treatment pauses could provide prolonged benefits for patients. Our results provide new insights into the contribution of miRNAs to drug-resistant NSCLC harboring EGFR-activating mutations and highlight their role as potential biomarkers and therapeutic targets.

## 1. Introduction

Non-small cell lung cancer (NSCLC) is the most prevalent type of lung cancer, accounting for approximately 85% of all cases and the leading cause of cancer-related deaths worldwide [1]. In 15–20% of NSCLC cases, mutations located in exon 19 and exon 21 of the epidermal growth factor receptor (EGFR) lead to constitutive activation, resulting in poor prognosis and short progression-free survival (PFS). EGFR tyrosine kinase inhibitors (TKIs) have significantly improved therapeutic options for patients with tumors harboring EGFR-activating mutations [2,3,4]. 

First-generation EGFR-TKIs, such as erlotinib and gefitinib, became the standard-of-care for NSCLC patients with EGFR-activating mutations and provided significant clinical benefits, including improved PFS and quality of life. However, their use was associated with relatively rapid resistance formation [2,3,4]. Second- and third-generation EGFR-TKIs, like afatinib and the current standard-of-care osimertinib, were designed to address resistance mechanisms caused by first-generation EGFR-TKI treatment [2,3,4]. Despite improved outcomes, resistance formation also occurs in response to third-generation EGFR-TKI treatment in a majority of patients within 9 to 12 months [3,5,6,7,8]. Intensive research efforts have revealed multiple resistance mechanisms, including C797S and T790M gatekeeper EGFR mutations, MET amplification, PIK3CA mutations, and epithelial-mesenchymal transition (EMT) [7,9,10].

EMT is a dynamic and reversible genetic program in which epithelial cells lose their apical-basal polarity and acquire traits typically associated with mesenchymal cells [11,12,13]. Moreover, EMT is associated with drug resistance, increased metastasis, and the acquisition of stem-like traits [13,14,15,16,17,18]. Emerging evidence has revealed that EMT is not a binary model but instead a hybrid epithelial/mesenchymal state [19,20,21]. EMT-hybrid cells can exhibit remarkable plasticity, enabling them to transition between epithelial and mesenchymal states in response to microenvironmental stimuli [22]. Circulating tumor cells (CTCs) are often associated with an EMT-hybrid state [23,24], emphasizing the urgency to develop therapeutics targeting this state.

MicroRNAs (miRNAs) are small non-coding RNAs that regulate gene expression by binding the 3′-untranslated regions (3′-UTR) of mRNA transcripts, resulting in transcript degradation and/or translational repression [25,26]. There is mounting evidence linking miRNAs to lung cancer, where they exert crucial roles in cellular transformation, functioning as either oncogenes or tumor suppressors [27,28]. Moreover, miRNAs have emerged as key regulators of EMT by targeting EMT transcription factors (EMT-TFs) belonging to the SNAIL, TWIST, and ZEB families [29,30,31,32,33,34], which act as molecular switches for EMT [35,36,37]. Additionally, miRNAs participate in the regulation of the tumor microenvironment when secreted in extracellular vesicles (EVs). 

EVs are small nanovesicles with a diameter of approximately 30–150 nm that envelope components, such as proteins, nucleic acids, and lipids. EV-derived miRNAs hold promising potential for diagnostic and prognostic applications [38,39,40,41,42] due to their stability and availability in sputum and plasma. Although numerous studies have aimed to identify appropriate miRNA candidates for diagnostic purposes [42,43,44,45,46], miRNAs are not yet diagnostically used in a clinical setting. Due to the heterogeneity of the resistance mechanisms, the understanding of specific miRNA fingerprints may be required to predict accurately the resistance status of tumors of individual patients. 

Here, we identified EMT-related miRNAs contributing to drug resistance in NSCLC by analyzing NSCLC cell lines adapted to 1st-, 2nd-, and 3rd-generation EGFR-TKIs.

## 2. Results

### 2.1. Drug Resistance Was Accompanied by EMT-Induced Phenotypic Changes and Increased Migration

**Figure 1:**
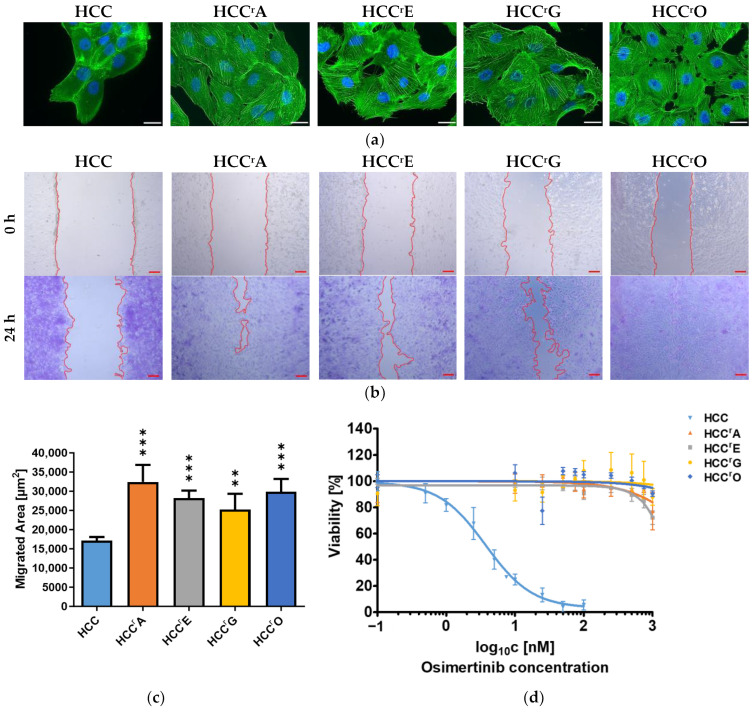
Resistance formation of the EGFR-mutant HCC4006 NSCLC cell line to EGFR-TKIs results in increased cell migration and cross-resistance to osimertinib: (**a**) Immunofluorescence staining of the actin cytoskeleton with phalloidin (green) revealed the introduction of actin stress fibers. Nuclei were stained with DAPI (blue). Scale bar: 25 µm. (**b**) Migration of cells was assessed by wound healing assay. Images were taken directly after the wound was mechanically introduced and after 24 h. Cells were fixed and stained with crystal violet solution for better visualization. Scale bar: 100 µm. (**c**) The extent of the area covered by the cells was analyzed with ImageJ. Results are displayed as means + SD with *n* = 4. A 1-way ANOVA with Dunnett’s post hoc test was used to test for statistically significant differences relative to the parental HCC4006 cell line. ∗∗ *p* < 0.01, ∗∗∗ *p* < 0.001 (**d**) Cells were treated with increasing concentrations of osimertinib for 72 h and then incubated with resazurin. Fluorescence was determined at 560 nm excitation and 590 nm emission. Cell viability was assessed by normalization to the DMSO-treated control and is shown as the mean ± SD of three biological replicates.

The NSCLC cell line HCC4006 was adapted to the EGFR-TKIs afatinib (HCC^r^A), erlotinib (HCC^r^E), gefitinib (HCC^r^G), and osimertinib (HCC^r^O) by continuous exposure to increasing drug concentrations. Resistance development was accompanied by significant changes in cell morphology, including the downregulation of cell-cell adhesion junctions, remodeling of the actin cytoskeleton, and the emergence of stress fibers (Figure 1a). Cell transformation was further associated with enhanced cell migration, as indicated by more rapid wound closure in wound healing assays (Figure 1b,c). Additionally, the proliferation of resistant cells was comparatively slower than that of the parental cell line (Supplementary Table S2). The T790M gatekeeper mutation was not the primary resistance mechanism, as all 1st and 2nd generation EGFR-TKI-resistant sublines were cross-resistant to the 3rd generation EGFR-TKI osimertinib (Figure 1d).

**Figure 2:**
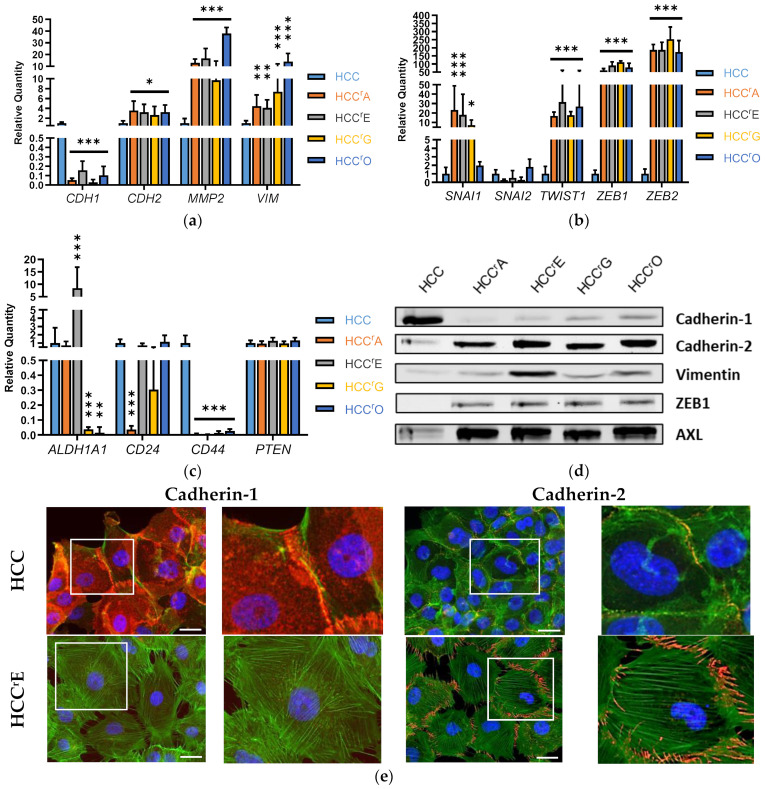
Epithelial-mesenchymal transition (EMT) is confirmed in all resistant sublines: (**a**) Gene expression of EMT markers, (**b**) EMT transcription factors, and (**c**) stem cell markers measured by qPCR. Results are displayed as the mean + SD of the relative quantity (displayed as ∆C_T_ in Figure S1) with *n* ≥ 3. A 1-way ANOVA with Dunnett’s post hoc test was used to test for statistically significant differences relative to the HCC4006 cell line, * *p* < 0.05, ** *p* < 0.01, *** *p* < 0.001. (**d**) Protein levels of the EMT-markers, ZEB1, and tyrosine-protein kinase receptor UFO (AXL) were measured by Western blot. A representative blot of three biological replicates is shown. (**e**) Immunofluorescence staining of cadherin-1 and cadherin-2 in parental and erlotinib-resistant HCC4006; DAPI (blue), actin (green), cadherin-1/cadherin-2 (red). White squares depict magnified areas. Scale bar: 25 µm.

We hypothesized that the cells had undergone EMT, which is often observed in EGFR-TKI-resistant cells [16,17]. To confirm this, we analyzed the mRNA and protein levels of EMT-marker and EMT-TF genes by qPCR, Western blotting, and immunofluorescence (Figure 2).

We observed downregulation of the epithelial marker *CDH1* (*n* = 3, *p* < 0.001) and a significant increase of the mesenchymal markers *MMP2*, *CDH2*, and *VIM* (Figure 2a), confirming that all resistant sublines had undergone EMT. The EMT-TFs *ZEB1/2* and *TWIST1* were uniformly upregulated in the resistant cells (*n* = 3, *p* < 0.001), but there was no change in *SNAI2* expression (Figure 2b). *SNAI1* expression was increased in HCC^r^O compared to HCC4006 but not in HCC^r^A, HCC^r^E, or HCC^r^G. 

We also analyzed the expression of cancer stem cell markers *ALDH1A1*, *CD24*, and *CD44*, which are often associated with EMT induction [47,48,49,50,51] (Figure 2c). However, there was no distinct expression pattern that would classify the resistant cells as cancer stem cells. *CD44* expression was significantly lower (>27-fold, *p* < 0.001) in the resistant cells, and *CD24* expression remained mostly unchanged except for HCC^r^A. *ALDH1A1* was overexpressed in HCC^r^E (23-fold, *p* < 0.001) but downregulated in HCC^r^G and HCC^r^O cells (>42-fold, *p* < 0.01). Moreover, transcriptional repression of tumor suppressor and EMT inducer phosphatase and tensin homolog (*PTEN*) [52,53,54] was not observed in the resistant cells. 

The analysis of the protein levels of EMT-markers and EMT-TFs by Western blot and immunofluorescent microscopy (Figure 2d,e), confirmed the downregulation of the epithelial marker cadherin-1 and the upregulation of the mesenchymal markers cadherin-2 and vimentin and of the EMT-TF ZEB1. In addition, protein levels of the tyrosine-protein kinase receptor UFO (AXL), which is often cited as an EMT inducer [55,56,57], were upregulated in all resistant cell lines. The replacement of cadherin-1 with cadherin-2 and its localization in filopodia in erlotinib-resistant cells was confirmed with immunofluorescence microscopy (Figure 2e). 

### 2.2. Differential Expression of EMT-Associated miRNAs Indicates ZEB1 as the Main Driver of EMT-Linked Drug Resistance

miRNAs have been shown to play a critical role in the post-transcriptional gene regulation of EMT. Therefore, we investigated miRNAs that have been implicated in the regulation of EMT-TFs to identify key EMT-related miRNAs that displayed similar changes in all resistant cell lines. We also decided to include the epithelial lung cell line 16HBE14o- as an untransformed control cell line. 

**Figure 3:**
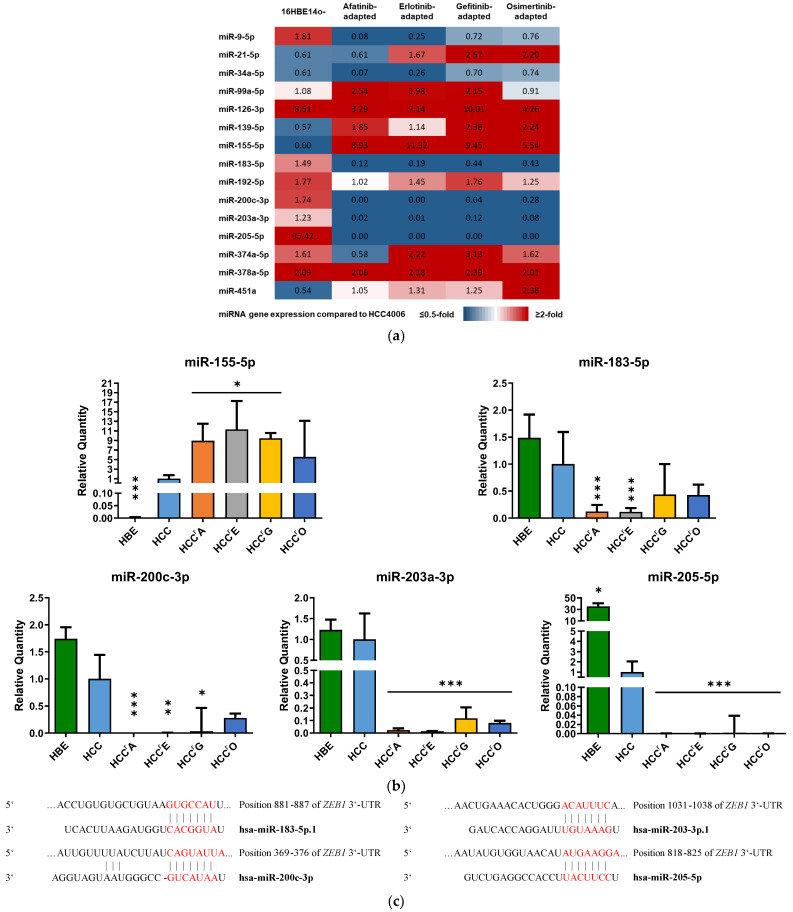
Expression of key EMT-regulating miRNAs in 16HBE14o- (untransformed), HCC4006 (NSCLC), and EGFR-TKI resistant HCC4006 sublines: miRNA levels were measured by qPCR and results (**a**) were displayed as a heatmap in a range from <0.5 to >2-fold or (**b**) shown as individual plots. Results are shown as the mean + SD of the relative quantity with *n* ≥ 3. A 1-way ANOVA with Dunnett′s post hoc test was used to test for statistically significant differences relative to the HCC4006 cell line, * *p* < 0.05, ** *p* < 0.01, *** *p* < 0.001. (**c**) Binding sites of miRNAs at the *ZEB1* 3′-UTR predicted by TargetScanHuman 8.0. Predicted consequential pairing (red) of target region (top) and miRNA (bottom).

We analyzed the abundance of 15 miRNAs that were found to be involved in the regulation of EMT [29,30,58,59]. The results are displayed as a heatmap (Figure 3a) with an expression fold change threshold ranging from ≤0.5 to ≥2. To be regarded as a relevant EMT-related miRNA, the levels of the miRNA needed to (1) exceed the defined threshold, (2) show a uniform expression in the resistant cell lines, and (3) a ≥2-fold expression increase compared with the parental and untransformed cell lines. Although most of the selected miRNAs showed differential expression in at least one resistant subline, only 5 miRNAs (miR-155-5p, miR-183-5p, miR-200c-3p, miR-203a-3p, miR-205-5p) met the criteria defined above (Figure 3a,b). Interestingly, the analysis of potential targets of these miRNAs using the miRNA target prediction tools TargetScanHuman 8.0 (Release September 2021) [60] and miRDB [61,62] identified *ZEB1* as a key target for miR-183-5p, miR-200c-3p, miR-203a-3p, and miR-205-5p (Figure 3c).

### 2.3. EMT-Associated miRNAs Were among the Highest-Ranked EV-Derived miRNAs Revealed by RNA-Sequencing

Subsequently, we aimed to validate whether the EMT-miRNA profile identified within the cells could also be verified in EVs released by the cells. We isolated EVs from the cell culture supernatants of HCC4006, HCC^r^E, HCC^r^A, and HCC^r^O using ultracentrifugation for the enrichment of the EVs in cell culture supernatants followed by the capturing of exosomes and other EVs using membrane affinity spin columns. EVs were characterized for size and size distribution by dynamic light scattering and scanning electron microscopy (Supplementary Figure S2a,b; Supplementary Tables S5 and S6). The exosome marker CD63 was used for the identification of exosomes by Western blotting (Supplementary Figure S2c). 

**Figure 4:**
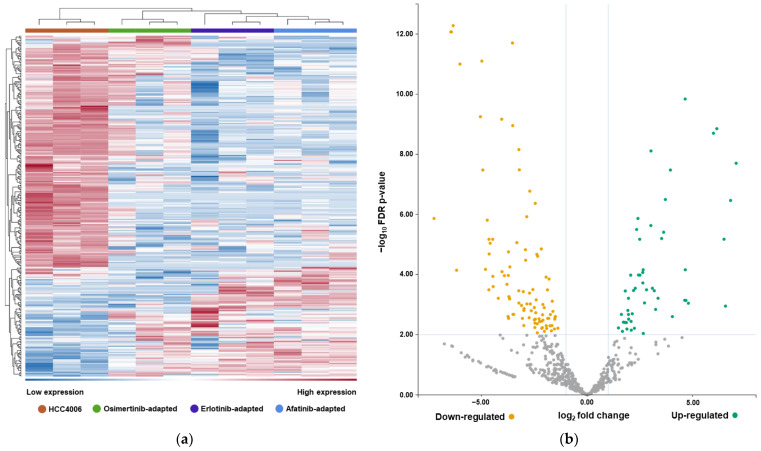

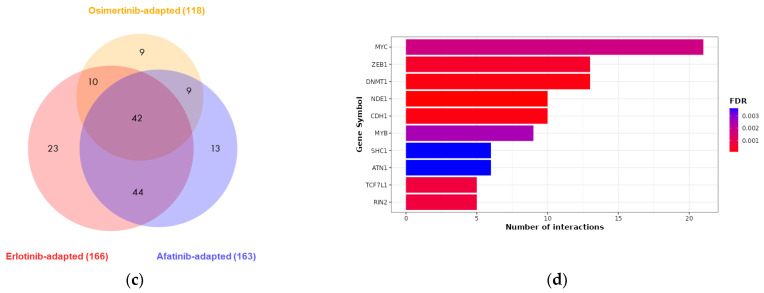
Sequencing of EV-derived miRNA reveals resistance-induced deregulation: (**a**) Clustered heatmap of EV-derived miRNA levels from HCC4006, HCC^r^O, HCC^r^E, and HCC^r^A; the *x*-axis shows the individual samples and the *y*-axis shows the miRNAs. (**b**) Volcano plot of the most strongly up- (green) and downregulated (yellow) miRNAs comparing the data from all resistant sublines merged into one dataset to HCC4006, log_2_ fold change < −2/> 2, log_10_ FDR < 2 (omitted shown in grey). (**c**) Venn diagram indicating uniformly deregulated miRNAs in the EVs derived from the resistant sublines, fold change < −2/> 2, FDR < 0.01. (**d**) Number of interactions between miRNAs and gene targets predicted by miRTarBase using the MIENTURNET algorithm.

Next, we sequenced the miRNAs from the affinity-captured EVs of parenteral and resistant HCC4006 (Figure 4). Erlotinib, afatinib and osimertinib-resistant sublines were analyzed to represent cells adapted to EGFR-TKIs from each generation. The majority of the analyzed miRNAs were downregulated in the resistant sublines (52 up, 97 down, fold change < −2/> 2, FDR < 0.01). We identified 42 miRNAs that were uniformly deregulated in all resistant sublines (Figure 4c). Target prediction analysis employing the MIENTURNET web tool [63] using the miRTarBase database [64] identified *MYC*, *ZEB1*, *DNMT1*, and *CDH1* (FDR < 0.002) among the top-ranked targets of these miRNAs. To determine the miRNAs that displayed the highest level of differential expression compared to the parental HCC4006 cell line, data from all resistant sublines were pooled into one dataset (Table 1).

Out of the ten most downregulated miRNAs detected in EVs, seven exhibited *ZEB1/2* as a predicted target, according to TargetScanHuman and miRDB, with nine out of ten inhibiting at least one EMT-associated gene. Moreover, the miR-200/429 family members were among the most strongly downregulated miRNAs. Noteworthy, the relative miRNA levels detected in EVs showed substantial similarities to the cellular miRNA expression patterns measured by qPCR.

### 2.4. Transfection of Erlotinib-Adapted HCC4006 (HCC^r^E) Cells with a miR-205-5p Mimic Partly Reversed EMT

**Figure 5:**
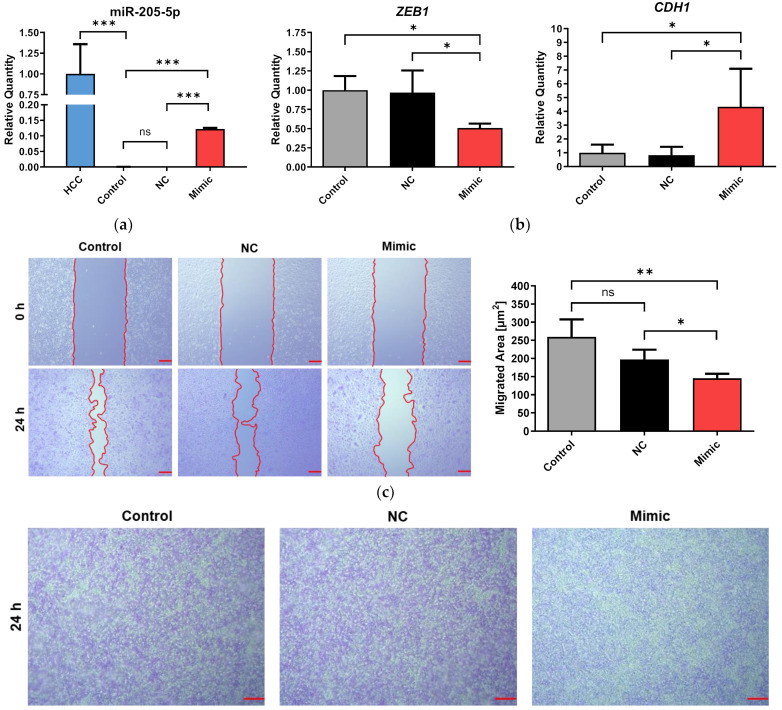

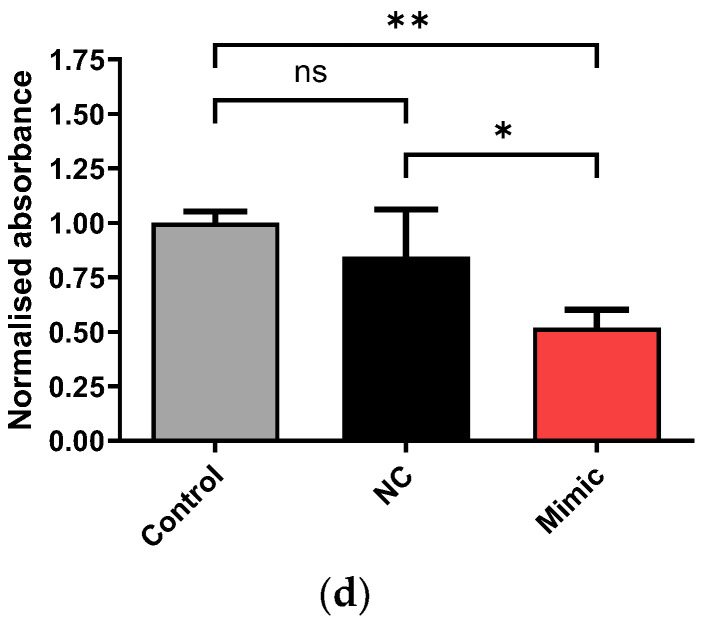
miR-205-5p transfection inhibits *ZEB1*, restores *CDH1,* and inhibits cell migration: Erlotinib-adapted HCC4006 (HCC^r^E) were either untreated (Control) or transfected with either 10 nM of a miR-205-5p mimic (Mimic) or 10 nM of a negative control siRNA (NC) for 48 h. (**a**) Validation of miR-205-5p mimic transfection. miR-205-5p expression in transfected and untransfected HCC^r^E- and HCC4006 (HCC) cells was measured by qPCR. (**b**) Gene expression in *ZEB1* and *CDH1* after miR-205-5p transfection in HCC^r^E cells measured by qPCR. (**c**) Wound healing migration assays after mimic transfection. Scale bar: 100 µm (**d**) Transwell migration assays after mimic transfection. Scale bar: 100 µm. Results are displayed as means + SD with *n* = 3. A 1-way ANOVA with Dunnett’s post hoc test was used to test for statistically significant differences relative to the untreated HCC^r^E cell line. An unpaired Student’s *t*-test was used to compare the NC and Mimic group, ns = not significant, * *p* < 0.05, ** *p* < 0.01, *** *p* < 0.001.

To investigate what effect the reversal of changes in the levels of EMT-associated miRNAs has on the resistant cells, HCC^r^E, which displayed the lowest miR-205-5p levels, were left untreated (Control) or transfected with either a miR-205-5p mimic (Mimic) or a negative control siRNA (NC). Transfection of 10 nM miR-205-5p mimic led to a 410-fold increase (*n* = 3, *p* < 0.001) of ectopic miRNA expression compared with the untreated and mock-transfected cells (Figure 5a). This was still well below the miR-205-5p expression of the parental HCC4006 cells, which was 3000-fold higher than in the untreated HCC^r^E cells. Reintroduction of the miRNA partly reversed EMT by significantly reducing the expression of *ZEB1* (2-fold, *n* = 3, *p* < 0.05) and restoring *CDH1* expression (4.3-fold, *n* = 3, *p* < 0.05) (Figure 5b). 

Next, we conducted wound healing and transwell migration assays to assess the effects of the miR-205-5p mimic on cell migration. Wound healing, as well as transwell migration, were both significantly inhibited by the mimic (*n* = 4, *p* < 0.01) (Figure 5c,d).

To gain insights into the progression of EMT during resistance formation to EGFR-TKI, we employed a stepwise adaptation approach osimertinib, which is currently the standard of care for NSCLC patients with tumors harboring EGFR-activating mutations. This allowed us to simulate the development of drug resistance and EMT induction. We analyzed the expression of EMT-TFs, EMT-markers, and EMT-miRNAs in osimertinib-adapted HCC4006 cells at seven adaptation levels (6.7, 12, 25, 50, 100, 250, 500 nM) to elucidate how the expression levels of EMT drivers evolved throughout the adaptation process.

**Figure 6:**
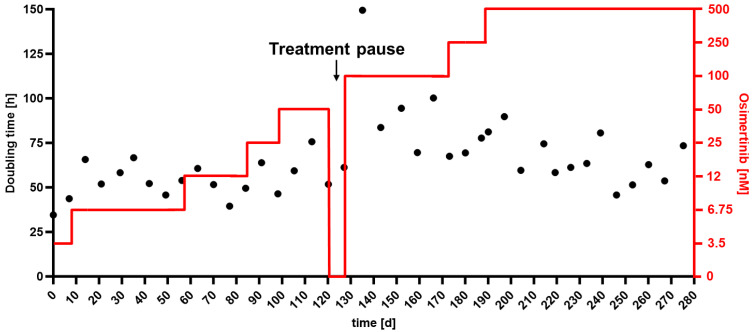
Stepwise adaptation of HCC4006 against 3rd generation EGFR-TKI osimertinib: HCC4006 cells were adapted to grow in increasing concentrations of osimertinib. Concentration was increased when doubling times plateaued in drug-tolerant cells. Adaptation was done in two major steps. After reaching 50 nM concentration, cells were cultured in inhibitor-free media for a week, then returned to osimertinib. Adaptation was completed after cells were able to grow in the presence of 0.5 µM osimertinib with a constant doubling time of ~60 h.

The adaptation was carried out as a continuous process in two major stages, from 3.5 to 50 nM and from 50 to 500 nM over the course of 278 days (Figure 6). After reaching the 50 nM adaptation level, the cells were cultivated in osimertinib-free media for one week to determine whether they would maintain the changes acquired during osimertinib adaptation. Cells were harvested for genetic characterization at the start of the subsequent adaptation level. Interestingly, upon continuing the adaptation process after the treatment pause, the cells displayed enhanced sensitivity against osimertinib, as seen by a substantial increase in doubling time (150 h).

**Figure 7:**
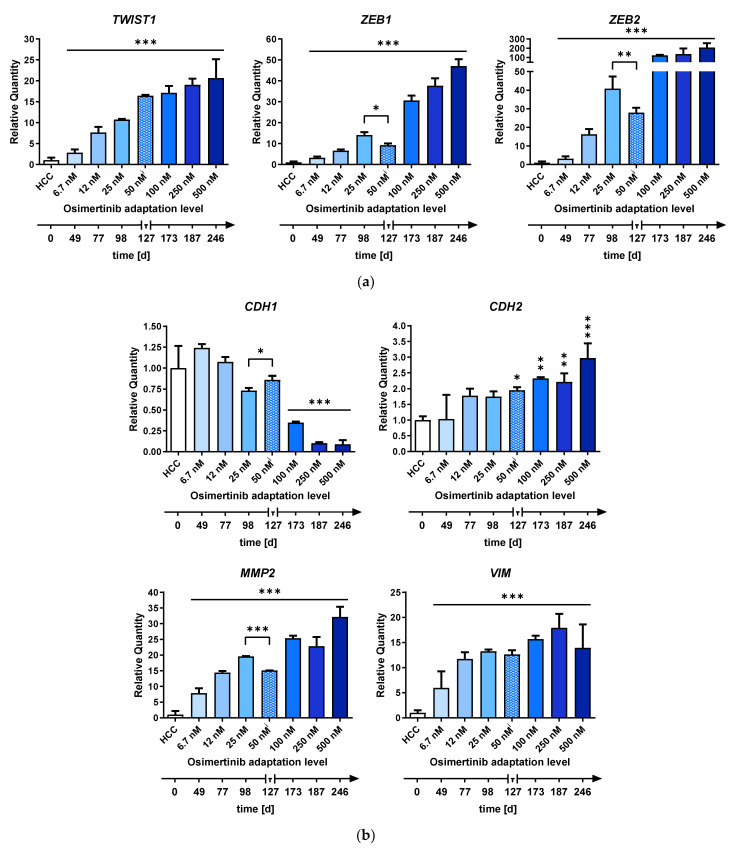

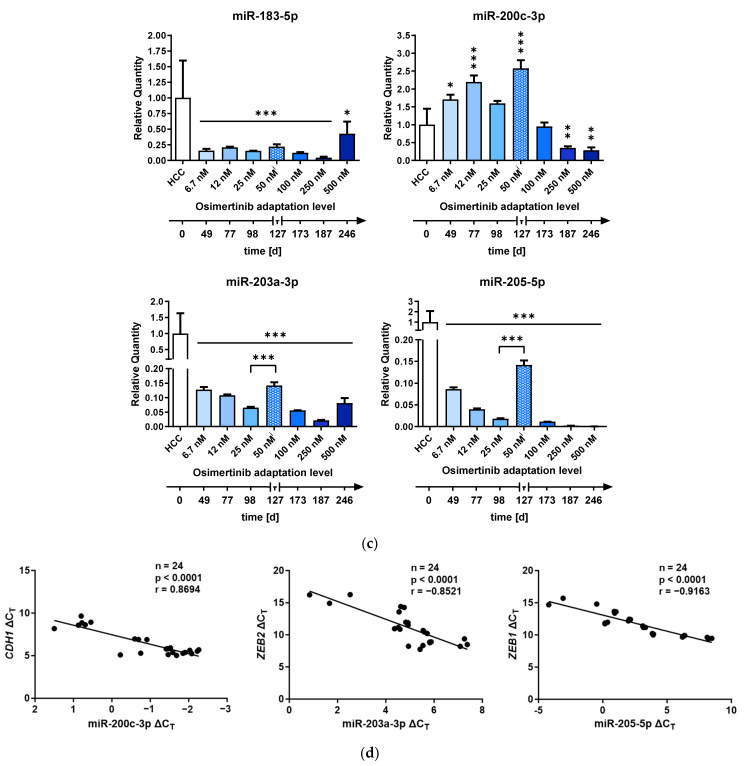
EMT-miRNA expression is closely correlated with the expression of EMT-TFs and EMT-markers: Expression of (**a**) EMT-markers, (**b**) EMT-TFs, and (**c**) EMT-miRNAs in HCC4006 grown in increasing concentrations of osimertinib as measured by qPCR. A treatment pause of 1 week was implemented after the 50 nM adaptation level was reached. Cells were harvested for characterization at the start of the subsequent adaptation level and after the treatment pause was completed (50 nM^i^). Results are displayed as the relative quantity as means + SD of three biological replicates. A 1-way ANOVA with Dunnett’s post hoc test was used to test for statistically significant differences relative to untreated HCC4006 cells. An unpaired Student’s *t*-test was used to compare the 25 nM and 50 nM**^i^** adaptation levels in cases of visible trend reversions, * *p* < 0.05, ** *p* < 0.01, *** *p* < 0.001. (**d**) Graphical representation of the Pearson correlation of the delta-C_T_ of EMT-miRNAs, EMT-TFs, and EMT-markers. r = Pearson correlation coefficient.

The expression of EMT-TFs increased over the course of the adaptation, reaching their maxima at the highest adaptation level (Figure 7a). A significant fold change increase (*TWIST1* = 2.8, *ZEB1* = 3.2, *ZEB2* = 3.1, *n* = 3, *p* < 0.001) was already reached at the lowest analyzed adaptation level (6.7 nM). Moreover, the expression pattern of EMT-marker genes displayed a similar trend (Figure 7b). The development of *MMP2* and *VIM* closely mirrored the expression pattern of *ZEB1* and *ZEB2*, exhibiting an early and substantial increase in expression (*MMP2* = 7.8, *VIM* = 6, *n* = 3, *p* < 0.001). In contrast, the expression of *CDH1* and *CDH2* only significantly exceeded the 2-fold threshold at the 100 nM adaptation level. 

Our next objective was to verify that the identified EMT-miRNAs drove the induction of EMT-TFs and the repression of *CDH1*. As expected, miR-183-5p, miR-203-3p, and miR-205-5p expression was dramatically reduced (>5-fold) even at the lowest adaptation level and continuously decreased during the adaptation process (Figure 7c). In contrast, miR-200c-3p expression first increased 2.5-fold but then sharply declined at the 100 nM adaptation level. When osimertinib was removed from the culture media for one week between the adaptation steps, there was a reversal of the observed trends. The expression of EMT-miRNAs miR-200c-3p, miR-203a-3p, and miR-205-5p increased significantly in the absence of osimertinib (*p* < 0.01) compared to the previous adaptation level (25 nM). In turn, the expression of the EMT-miRNA target genes *ZEB1* and *ZEB2* declined significantly (*p* < 0.001). Removal of osimertinib also had a significant effect on the expression of the EMT-markers *CDH1* (*p* < 0.05) and *MMP2* (*p* < 0.001). However, *TWIST1*, *CDH2,* and *VIM* were not significantly affected. There was a close correlation between miR-200c-3p ΔC_T_ and *CDH1* ΔC_T_ (Pearson’s r = 0.8694, *n* = 24, *p* < 0.0001), while miR-203-3p and miR-205-5p showed strong correlations with *ZEB1* (r = −0.8521, *n* = 24, *p* < 0.0001; r = 0.9028, *n* = 24, *p* < 0.0001) and *ZEB2* (r = −0.8157, *n* = 24, *p* < 0.0001; r = −0.8872, *n* = 24, *p* < 0.0001) (Figure 7d).

## 3. Discussion

The emergence of drug resistance typically after 9–12 months of treatment limits the success of osimertinib as a first-line therapeutic for EGFR-mutant NSCLC [5,65,66,67]. EMT contributes to drug resistance and is often observed in NSCLC patients with acquired EGFR-TKI resistance [5,16,65,66,67]. However, the induction of EMT during treatment and the role of resistance-induced miRNAs remain poorly understood. Therefore, the objective of this study was to investigate the role of miRNAs in the initiation and sustenance of EMT in EGFR-TKI-induced drug resistance. 

To accomplish this, we established a cell panel comprising HCC4006 sublines that were individually adapted to various EGFR-TKIs. We selected EGFR-TKIs that are currently in use for patient treatment. The resistant sublines uniformly underwent EMT, which was confirmed by comprehensive EMT-marker and EMT-TFs expression analyses. Transitioning from an epithelial to a mesenchymal phenotype in EGFR-TKI resistant sublines was associated with increased cell mobility and migration, probably as a consequence of the remodeling of the actin cytoskeleton and the introduction of actin stress fibers. However, these changes were, in agreement with findings by others [65,68], accompanied by a reduced cell doubling time. This demonstrated that the HCC4006 cell line is a reliable in-vitro model to simulate EGFR-TKI resistance-induced EMT. Furthermore, all cell lines remained resistant to osimertinib, which indicates that they had not acquired the common T790M gatekeeper mutation [66,67]. Previous studies have often focused on a single EGFR-TKI, which may limit their generalisability [69,70]. In addition, concentrating solely on one specific resistant cell line could lead to confounding results and erroneous conclusions. Therefore, adopting a broader approach when using in-vitro cell models emulating drug resistance to identify reoccurring mechanisms is of utmost importance, as shown by this study in the case of cancer stem cell (CSC) markers. Our findings suggest that the expression patterns of the common CSC markers *ALDH1A1* (high), *CD24* (low), and *CD44* (high) did not indicate a CSC phenotype of the resistant sublines. The use of these markers has been controversial in the literature [48,50,51,71,72], but they are often used due to the lack of better alternatives. Further research is necessary to discover markers or combinations thereof that can accurately identify CSCs in in-vitro NSCLC models and patients with high sensitivity. 

Moreover, our cell line panel analysis revealed that TWIST1, ZEB1, and ZEB2 were the predominant EMT-TFs responsible for maintaining the EMT phenotype in drug-resistant HCC4006 cells, whereas SNAI2 showed no significant involvement. SNAI1 was not uniformly deregulated in all resistant cells and might therefore represent a less universal drug target than TWIST1 or ZEB1/2. These results support previous findings reported by other authors [31,37,73,74,75]. In contrast, the commonly cited miRNA target PTEN [52,54,76,77,78] was not deregulated in this cell model. Interestingly, we observed an upregulation of AXL in the resistant cells, which has previously been proposed as a potential resistance mechanism [56,79,80].

In our study, we identified EMT-associated miRNAs that were uniformly deregulated in drug-resistant cells. Global miRNA expression was reduced in the resistant cells, consistent with other reports [81,82]. qPCR analysis and RNA-sequencing revealed that miR-183-5p, the miR-200/429 family, miR-203a-3p, and miR-205-5p were closely associated with EMT in drug resistance. These miRNAs and their likely target *ZEB1* have previously been linked to drug resistance [54,83,84,85,86,87,88]. Additionally, a miRNA target enrichment analysis identified *MYC* and *DNMT1* as main targets, which have also been proposed as resistance factors in other studies [89,90,91,92,93,94,95,96,97]. Furthermore, we noticed substantial similarities in the miRNA levels between cells and EVs, indicating that EV-derived miRNAs may be used in liquid biopsies for the monitoring of the resistance status. Their application as potential biomarkers for the identification of EMT-positive, drug-resistant tumor cells should therefore be further investigated, considering that not all findings from in-vitro resistance models translate into the clinics [98].

The transfection of a miR-205-5p mimic partly reversed EMT by inhibiting *ZEB1* and restoring *CDH1* expression. Additionally, the miR-205-5p mimic reduced cell migration in wound healing and transwell assays, highlighting the therapeutic potential of miRNAs as drug targets [99,100,101,102]. However, given the extensive genetic alterations and cancer cell heterogeneity induced by drug resistance [42,43,44,45], a potential strategy for achieving a complete reversal of EMT and restoring therapeutic efficacy might involve the targeting of multiple miRNAs or the simultaneous modification of their expression levels with demethylating agents [22,93].

**Figure 8:**
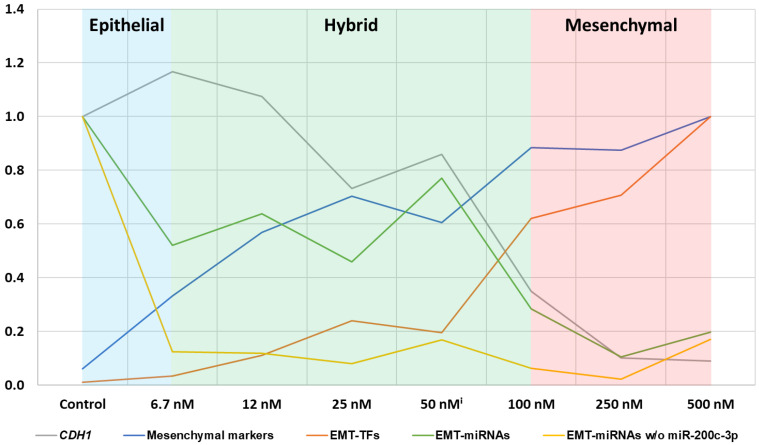
Expression patterns provide insights into EMT states in osimertinib-adapted HCC4006: Relative Expression data from Figure 7 is shown to visualize the trends of *CDH1*, mesenchymal markers *MMP2*, *CDH2*, and *VIM*; EMT-TFs *TWIST1*, *ZEB1*, and *ZEB2*; and EMT-miRNAs miR-183-3p, miR-200c-3p, miR-203a-3p, and miR-205-5p. Expression was normalized to the maximum fold change. A treatment pause of 1 week was implemented after the 50 nM adaptation level was reached. The cells were harvested for genetic characterization after the treatment pause was completed (50 nM^i^).

Through the analysis of the stepwise HCC4006 cell line adaptation to osimertinib, we revealed a close association between the loss of key miRNAs and the upregulation of EMT-TFs, leading to changes in EMT-marker expression. Our findings also showed that cellular reprogramming commenced at low doses and after a short treatment duration but intensified with escalating osimertinib concentrations. Consequently, low drug concentrations achieved in less perfused regions of the tumor might be sufficient to induce EMT reprogramming, generating drug-tolerant cells. Noteworthy, the downregulation of miR-183-5p, miR-203a-3p, and miR-205-5p expression was followed by an increase in *ZEB1/2* and *TWIST1* expression, with miR-200c-3p being lost only at higher osimertinib concentrations. Expression of the EMT-markers *CDH1* and *CDH2* exceeded the 2-fold threshold at higher adaptation levels, similarly to miR-200c-3p, suggesting a coordinated regulatory mechanism. Consequently, solely classifying tumors in patients based on epithelial or mesenchymal markers is inadequate [103,104,105,106]. The observed expression patterns of EMT-markers, EMT-TFs, and EMT-miRNAs during osimertinib adaptation provide valuable insights into the transition from an epithelial to a hybrid state (Figure 8), which is associated with increased invasion and migration capabilities [19,21,105,106,107]. Our results suggest that miRNA fingerprints could be reliable diagnostic markers to detect the early onset of EMT and transition from the epithelial to the hybrid state.

Additionally, circulating tumor cells, which have the potential to form distant metastases, often co-express these markers [24,108]. Therefore, identifying novel treatment strategies to specifically target hybrid cells remains a top priority and should be pursued with rigorous research efforts and clinical trials to improve treatment outcomes for patients. Temporally relieving the cells of the therapeutic pressure reversed transition trends, particularly evident at the miRNA level, where we observed a remarkable increase in expression. Therapy regimens with the implementation of treatment pauses, as previously employed [109,110], could potentially delay the onset of EMT and prolong the benefits for patients from these drugs. However, further validation of this hypothesis through clinical investigations under real-world conditions is necessary.

## 4. Materials and Methods

### 4.1. Chemicals

All chemicals were of analytical grade or higher. Primary antibodies and AlexaFluor^®^-conjugated secondary antibodies were purchased from Cell Signaling Technology (Leiden, The Netherlands) or ThermoFisher (ThermoFisher Scientific, Darmstadt, Germany). IRDye^®^-conjugated secondary antibodies were purchased from LI-COR Biosciences (Bad Homburg, Germany). A full list of used antibodies and dilutions is provided in Supplementary Table S1.

### 4.2. Cell Culture

The NSCLC cell line HCC4006 (HCC) was purchased from ATCC (Manassas, VA, USA). The resistant sublines HCC4006^r^AFA^0.1^ (HCC^r^A), HCC4006^r^ERLO^0.5^ (HCC^r^E), HCC4006^r^GEFI^0.5^ (HCC^r^G) were established as previously described [111] and derived from the Resistant Cancer Cell Line (RCCL) collection (https://research.kent.ac.uk/industrial-biotechnology-centre/the-resistant-cancer-cell-line-rccl-collection/; accessed on 18 August 2023). The resistant subline HCC4006^r^OSI^0.5^ (HCC^r^O) was established by adapting parental chemo-sensitive HCC to grow in the presence of osimertinib by continuous exposure to increasing concentrations, as described before [111]. Once drug tolerance reached 0.5 µM, the cells were continuously exposed to 0.5 µM osimertinib throughout the cultivation process. The corresponding doubling times and IC_50_ values for the parental cell line and their resistant sublines are provided in Supplementary Table S2. 

The cell line 16HBE14o- (HBE) was gifted by Jan-Peter Hildebrand (University of Greifswald). 

All cell lines were cultured in DMEM high glucose medium (Sigma-Aldrich Chemie GmbH, Munich, Germany) supplemented with 10% fetal bovine serum (FBS) (PAN Biotech GmbH, Aidenbach, Germany) and the respective EGFR-TKI at 37 °C in a humidified 5% CO_2_ atmosphere. Cell count and viability were assessed by electronic current exclusion (CASY, Omni Life Sciences GmbH & Co KG, Bremen, Germany). Cells were routinely tested for mycoplasma contamination by quantitative real-time PCR (qPCR).

### 4.3. Cell Proliferation Analysis

Cells were seeded into 96-well plates (10,000 cells/well for HCC, 4000 cells/well for the resistant cell lines) and incubated with inhibitors solubilized in a maximum of 0.1% DMSO for 72 h. Cells were then further incubated with 4 mg/mL resazurin for 3 h. Fluorescence of the metabolite resorufin was determined after 3 h at 560 nm excitation and 590 nm emission wavelength (Infinite 200 PRO, Tecan Group AG, Männedorf, Switzerland). 

### 4.4. RNA Extraction and cDNA Synthesis

All reverse transcription (RT) and PCR reagents were purchased from New England Biolabs (Frankfurt/Main, Germany) unless stated otherwise. Total RNA was prepared using the Monarch Total RNA miniprep Kit according to the manufacturer’s recommendations. 

Isolated RNA was treated with DNase I (ThermoFisher Scientific, Darmstadt, Germany) for 20 min at 37 °C to remove any residual genomic DNA. After adding 5 mM EDTA, enzyme inactivation was carried out for 10 min at 65 °C. Following this step, the purification process was performed using the Monarch Total RNA miniprep Kit according to the manufacturer’s recommendations. For mRNA analysis, RNA was converted to cDNA using the LunaScript RT Supermix Kit according to the manufacturer’s instructions. 

cDNA synthesis and analysis of miRNA was adapted from [112]. Briefly, 100 ng of RNA in a final volume of 10 µL including 2 µL of LunaScript RT Supermix Kit Primer-free, 0.1 mM of ATP, 1 µM of universal RT-primer, and 1 unit of poly(A) polymerase was incubated at 42 °C for 1 h followed by enzyme inactivation at 95 °C for 5 min. 

### 4.5. Quantitative Real-Time PCR

All primers were purchased from either Sigma-Aldrich (Sigma-Aldrich Chemie GmbH, Munich, Germany) or ThermoFisher (ThermoFisher Scientific, Darmstadt, Germany) Primer sequences and efficiencies are provided in Supplementary Table S3. qPCR was carried out in a 7 µL total volume with 0.14 µL of cDNA, 250 nM of each primer, and 3.5 µL Luna Universal qPCR Master Mix. Cycling conditions were 95 °C for 60 s, followed by 45 cycles of 95 °C for 15 s and 60 °C for 30 s. A melting curve analysis (60 °C to 95 °C) was performed to ensure specificity in the amplification. All qPCR experiments were performed on a QuantStudio^®^ 12K Flex (ThermoFisher Scientific, Darmstadt, Germany) with fast ramp speed. NormFinder [113] and BestKeeper [114] algorithms were employed to validate *GAPDH* and *ACTB* as suitable reference genes for mRNA, while miR-544a and snRNA *RNU6* were identified as appropriate reference miRNA/snRNA for miRNA. Samples were measured in triplicates, and the resulting data was analyzed by the comparative ΔΔC_T_ method. Statistical tests and Pearson correlation were performed on the raw ΔC_T_ data sets.

### 4.6. Western Immunoblotting

We seeded 5 × 10^5^ cells in 6-well plates and lysed them with 100 µL lysis buffer (50 mM Tris pH 7.6, 100 mM NaCl, 5 mM EDTA, 0.2 mM sodium vanadate, 1% Triton^®^ X-100). Next, 30 µg protein or 10 µL of lysed EVs were separated on 10–12% polyacrylamide gels, and proteins were subsequently transferred to nitrocellulose membranes through electroblotting (6 mA/cm^2^) for 1.25 h. Membranes were blocked with 5% milk powder in TBST buffer (Tris 2.42 g/L, NaCl 8.5 g/L, Tween-20 0.1%) for 2 h at room temperature. Antibody dilutions and incubations were performed according to the manufacturer’s recommendations. Membranes were analyzed with Odyssey imaging system (Licor Biosciences GmbH, Bad Homburg, Germany). Signal intensities were normalized to the total protein amount visualized by Ponceau S staining.

### 4.7. Immunofluorescent Microscopy

We seeded 8 × 10^4^ cells on 18 mm^2^ cover slips (Carl Roth GmbH & Co. KG, Steinheim, Germany) and grew them overnight. Cells were then fixed with 4% paraformaldehyde, permeabilized with 0.1% Triton^®^ X-100 and blocked with 3% BSA. Primary antibody incubation was done overnight at 4 °C and subsequent secondary antibody staining for 1 h at room temperature. Nuclei were stained with DAPI (0.5 µg/mL) and the actin cytoskeleton with Phalloidin-AlexaFluor^®^488. Samples were visualized with a Leica DMi8 fluorescent microscope (Leica Microsystems GmbH, Wetzlar, Germany).

### 4.8. RNA Interference

miR-205-5p mimic and mock miRNA control (NC) were purchased from Sigma-Aldrich (Sigma-Aldrich Chemie GmbH, Munich, Germany). The sequence of the miRNA mimic and mock miRNA control are provided in Supplementary Table S4. Sense and antisense strands were annealed by mixing at an equal molar concentration and heating at 95 °C (Thermocycler PCR W, Analytik Jena GmbH+Co. KG, Jena, Germany) for 5 min, followed by gradually cooling to room temperature. Next, 1 × 10^5^ cells were transfected with 10 nM negative control mimic (NC), 10 nM miR-205-5p mimic (Mimic) or left untreated (Control) for 48 h using Lipofectamine RNAiMAX (ThermoFisher Scientific, Darmstadt, Germany) according to the manufacturer’s recommendations.

### 4.9. Wound Healing Migration Assay

We seeded 1 × 10^5^ cells in a 12-well plate and grew them overnight. The confluent monolayer was scratched with a 100 µL tip and washed with PBS. Cells were incubated with 5 mM N-hydroxyurea to inhibit cell proliferation. After 24 h, cells were fixed with 70% ethanol for 30 min and stained with 0.01% crystal violet in deionized water. Images were taken (Primo Vert, Carl Zeiss Microscopy GmbH, Wetzlar, Germany) immediately and 24 h after the scratch was made. The area covered by the cells through migration was determined by manually analyzing the cell-free area with ImageJ [115]. The migrated area was calculated by subtracting the cell-free area measured at the end of the experiment from the cell-free area measured directly after the scratch was made. 

### 4.10. Transwell-Migration Assay

After miR-205-5p mimic transfection, 1 × 10^5^ cells were seeded in the upper chamber of a 24-transwell plate (Sarstedt AG & Co. KG, Nümbrecht, Germany) with 8.0 µm pores containing serum-free medium. The lower chamber was filled with a complete medium containing 10% FBS. After 24 h, cells on the upper surface of the membrane were removed with a cotton tip. Migrated cells on the lower surface were fixed with 70% ethanol and stained with 0.1% crystal violet in deionized water. After extensive washing with deionized water, images were taken (Primo Vert, Carl Zeiss Microscopy GmbH, Wetzlar, Germany), and crystal violet was dissolved with 100% methanol, and the absorption analyzed (Infinite 200 PRO, Tecan Group AG, Männedorf, Switzerland) at 590 nm. 

### 4.11. miRNA Isolation from Extracellular Vesicles

Cells were cultivated on five T150 dishes until they reached 70–80% confluency. Cells were then washed twice with PBS and incubated for 48 h with media supplemented with 5% exosome-depleted FBS. Exosome-depleted media was obtained by ultracentrifugation (Optima XPN-80/L7-65, Beckman Coulter GmbH, Krefeld, Germany) of media supplemented with 20% FBS at 110,000× *g* for 18 h at 4 °C and subsequent dilution as described before [116]. EV-containing cell culture supernatant (CCS) was carefully collected without disturbing the cells and centrifuged at 4000× *g* for 30 min at 4 °C to pellet cells. Cells were then washed with PBS and trypsinized to assess cell count and viability by electronic current exclusion. Only experiments with a cell viability value of >90% were considered acceptable for subsequent isolation. EV-containing CCS was then filtered through a 0.2 µm polyethersulfone (PES) filter (Sartorius AG, Göttingen, Germany) to retain cell debris and large vesicles. The filtered CCS was stored for not longer than 48 h at 4 °C before subsequent processing. Concentration of EV-containing CCS was done by ultracentrifugation at 110,000× *g* for 130 min at 4 °C. EVs and miRNAs from the EVs were isolated from the concentrated CCS using the exoRNeasy^®^ Midi Kit (Qiagen, Hilden, Germany) according to the manufacturer’s instructions. Briefly, the EV-enriched CCS was mixed with EV-binding solution (1:1) and placed on an EV-binding column. After binding and subsequent washing, the EVs were lysed with the use of Qiazol lysis buffer. After Qiazol-chloroform-extraction, the RNA-containing fraction was mixed (1:2) with 100% ethanol and placed on an RNA-binding column for purification. After all washing steps were concluded, the RNA was eluted with 14 µL nuclease-free water, and a 1 µL aliquot was used for concentration measurement with the Qubit™ microRNA Assay Kit (ThermoFisher Scientific, Darmstadt, Germany) All samples were immediately stored at −80 °C. 

### 4.12. Size and Size Distribution of Extracellular Vesicles 

EVs from the concentrated EV-containing CCS were analyzed by dynamic light scattering (DLS) and scanning electron microscopy (SEM). DLS measurements were performed using a Zetasizer Nano ZS (Malvern Pananalytical GmbH, Kassel, Germany) equipped with a 633-nm He-Ne laser and operating at an angle of 173°. EVs were diluted 1:100 to a total of 500 µL in PBS filtered through a 100 kDa cut-off filter and loaded into a disposable cuvette for particle size measurement. The particle size and size distribution were measured as cumulant (Z-Average) size and polydispersity index (PdI). Background measurements were done to ensure that the results were reliable. Data were acquired and analyzed using Zetasizer Software (V7.13) (Malvern Pananalytical GmbH, Kassel, Germany). All the experiments were performed in triplicates. 

SEM was used for single vesicle analysis. Immediately after isolation, EV samples were fixed with 2% glutaraldehyde in PBS for 90 min. Next, 5 µL of fixed EVs were mounted on a clean cover slip and dried overnight at 4 °C. The fixed Evs were then treated with 30%, 50%, 70%, 80%, 95%, and 100% ethanol sequentially for 10 min each. Samples were then dried in a desiccator over desiccated silica gel. The samples were sputtered (Mini Sputter Coater SC7620, Judges House, Lewes Road, Laughton, UK) with a 2–5 nm gold-palladium coat with argon plasma for 3 × 90 s at 18 mA. Sputtered samples were kept in the desiccator until analysis. SEM (Phenom G1, ThermoFisher Scientific, Dreieich, Germany) was performed under low-beam energy (5–10 kV).

### 4.13. RNA-Sequencing and Analysis

We used 100 ng purified RNA (if available; for HCC^r^O-2: 54.6 ng; HCC-3: 80.2 ng; HCC-2: 93.7 ng) as the starting material to prepare miRNA libraries using the QIAseq miRNA UDI Library Kit (Qiagen, Hilden, Germany) and QIAseq miRNA 12 Index Kit IL UDI (Qiagen) according to the manufacturer’s instructions. Briefly, adapters and primers were employed undiluted, and library amplification was performed with 16 cycles. Generated miRNA libraries were quality-controlled with Qubit dsDNA-HS-Assay-Kit (ThermoFisher Scientific, Darmstadt, Germany) and Agilent Bioanalyzer 2100 High Sensitivity DNA Kit (Agilent Technologies, Waldbronn, Germany) and all libraries passed quality criteria. Library concentrations were normalized to 4 nM and pooled prior to sequencing. Single-end sequencing with 72 bp and dual index sequencing of 10 bp each was performed on an Illumina NextSeq2000 (Illumina, San Diego, CA, USA). Processing, read mapping, and differential expression analysis of the resulting FASTQ files were performed with the QIAGEN RNA-seq Analysis Portal 4.0 (Qiagen, Aarhus, Denmark) using the miRNA analysis workflow. Target prediction analysis of significantly deregulated miRNAs (FDR < 0.01) was conducted using the MIENTURNET web tool [63] and the miRTarBase database [64].

### 4.14. Statistical Analysis

All experiments were carried out in triplicates unless stated otherwise. Data are presented as the mean + standard deviation (SD). Data were analyzed for statistical significance using an F-test to determine equal variances and an unpaired Student’s *t*-test or Welch’s *t*-test for single comparisons. One-way analysis of variance analysis (ANOVA) with Dunnett’s post hoc test were used for multiple comparisons on one dataset. A *p*-value of <0.05 was considered statistically significant. Correlations were analyzed using Pearson linear regression analysis. Statistical tests and Pearson linear regression analysis were performed using the GraphPad Prism Version 5.02 software (GraphPad Software Inc., San Diego, CA, USA).

## 5. Conclusions

In conclusion, our study highlights the crucial role of miRNAs and their target *ZEB1* in driving EMT in EGFR-TKI drug resistance and their potential as biomarkers and therapeutic targets. Temporally relieving cells of osimertinib demonstrated promising effects on miRNAs in EMT reversal, indicating prolonged benefits by incorporating treatment pauses to delay EMT onset and improve therapeutic efficacy. Overall, targeting hybrid cells and addressing the challenges of drug resistance through rigorous research and clinical trials remain imperative for advancing NSCLC treatment.

## Figures and Tables

**Table 1:** The top 10 differentially regulated miRNAs in HCC4006 vs. its EGFR-TKI-adapted sublines.

Gene ID	Fold Change	FDR *p*-Value	*p*-Value	Up-/Down- Regulation
hsa-miR-205-5p	−151.9	1.37 × 10^6^	5.34 × 10^8^	Down
hsa-miR-200b-3p	−87.6	8.51 × 10^13^	4.07 × 10^15^	Down
hsa-miR-203a-3p	−85.7	8.51 × 10^13^	3.10 × 10^15^	Down
hsa-miR-200a-5p	−81.0	5.27 × 10^13^	8.40 × 10^16^	Down
hsa-miR-655-3p	−72.4	7.23 × 10^5^	5.53 × 10^6^	Down
hsa-miR-375-3p	−64.9	9.99 × 10^12^	9.56 × 10^14^	Down
hsa-miR-432-5p	−33.1	5.63 × 10^10^	7.19 × 10^12^	Down
hsa-miR-431-3p	−31.6	7.96 × 10^12^	6.35 × 10^14^	Down
hsa-miR-429	−30.6	3.35 × 10^8^	9.61 × 10^10^	Down
hsa-miR-2276-3p	−28.1	6.74 × 10^5^	4.84 × 10^6^	Down
hsa-miR-99a-5p	133.2	1.99 × 10^8^	4.76 × 10^10^	Up
hsa-miR-1246	111.0	3.44 × 10^7^	1.15 × 10^8^	Up
hsa-miR-767-5p	93.8	1.13 × 10^3^	1.63 × 10^4^	Up
hsa-miR-1290	89.5	6.71 × 10^6^	3.32 × 10^7^	Up
hsa-miR-4508	70.6	1.41 × 10^9^	2.48 × 10^11^	Up
hsa-miR-9901	63.2	2.00 × 10^9^	3.83 × 10^11^	Up
hsa-miR-4497	27.8	9.06 × 10^4^	1.24 × 10^4^	Up
hsa-miR-4455	25.6	7.32 × 10^4^	9.58 × 10^5^	Up
hsa-miR-99a-3p	25.1	6.96 × 10^5^	5.22 × 10^6^	Up
hsa-miR-3182	25.0	1.45 × 10^10^	1.62 × 10^12^	Up

## Data Availability

The data presented in this study will be made available upon request. All image files are made available in high resolution in the Supplementary Files.

## Supplementary Materials

The following supporting information can be downloaded at: https://www.mdpi.com/article/10.3390/ijms241914742/s1.
