# Supplementary material for: Loss of Key EMT-Regulating miRNAs Highlight the Role of ZEB1 in EGFR Tyrosine Kinase Inhibitor-Resistant NSCLC
Source: Int J Mol Sci. 2023 Sep 29;24(19):14742. doi: 10.3390/ijms241914742 (PMC10573279; doi:10.3390/ijms241914742)
Supplement: Supplementary file 1 [file ijms-24-14742-s001.zip › Supplementary Materials.pdf]

Supplementary Materials

Loss of Key EMT-Regulating miRNAs Highlight the Role of ZEB1 in EGFR Tyrosine Kinase Inhibitor-Resistant NSCLC

Linus Gohlke, Ahmad Alahdab, Angela Oberhofer, Karolina Worf, Stefan Holdenrieder, Martin Michaelis, Jindrich Cinatl Jr., Christoph A. Ritter

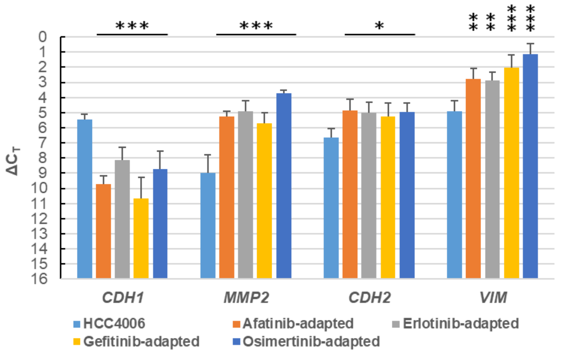

(**a**)

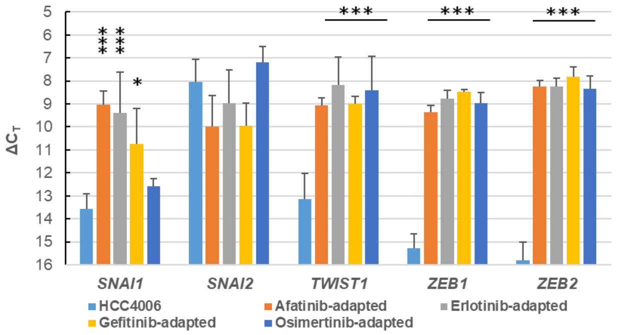

(**b**)

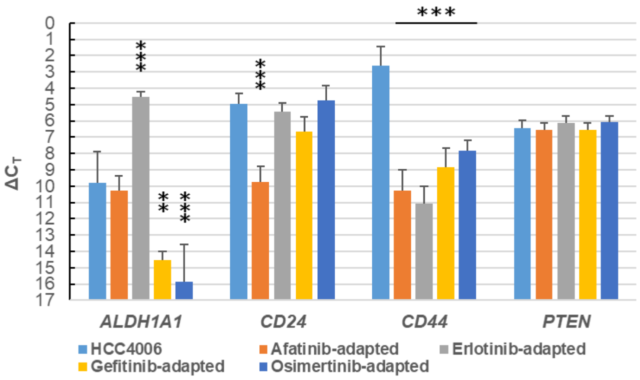

(**c**)

Supplementary Figure S1. Epithelial-mesenchymal transition is confirmed in all resistant cell lines: (a) Gene expression of EMT-markers, (b) EMT transcription factors, and (c) stem cell markers measured by qPCR. Results are displayed as the mean + SD of the delta-C_T with $n \geq 3$. A 1-way ANOVA with Dunnett's post hoc test was used to test for statistically significant differences relative to the parenteral HCC4006 cell line, * $p < 0.05$, ** $p < 0.01$, *** $p < 0.001$.

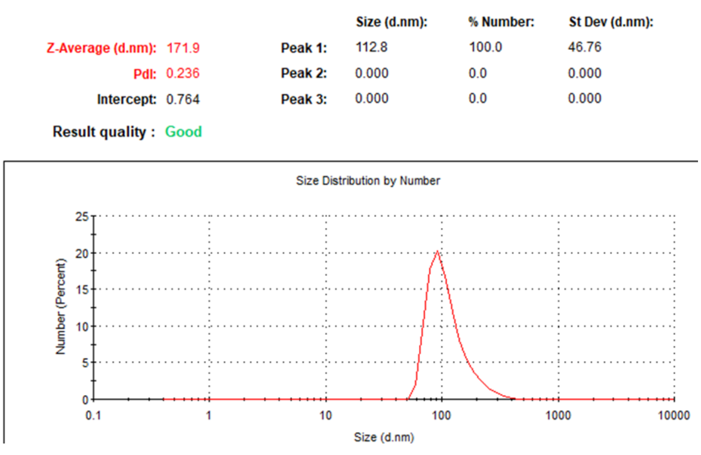

(a)

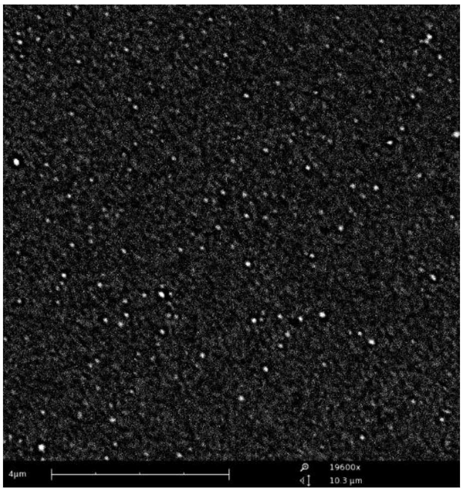

(b)

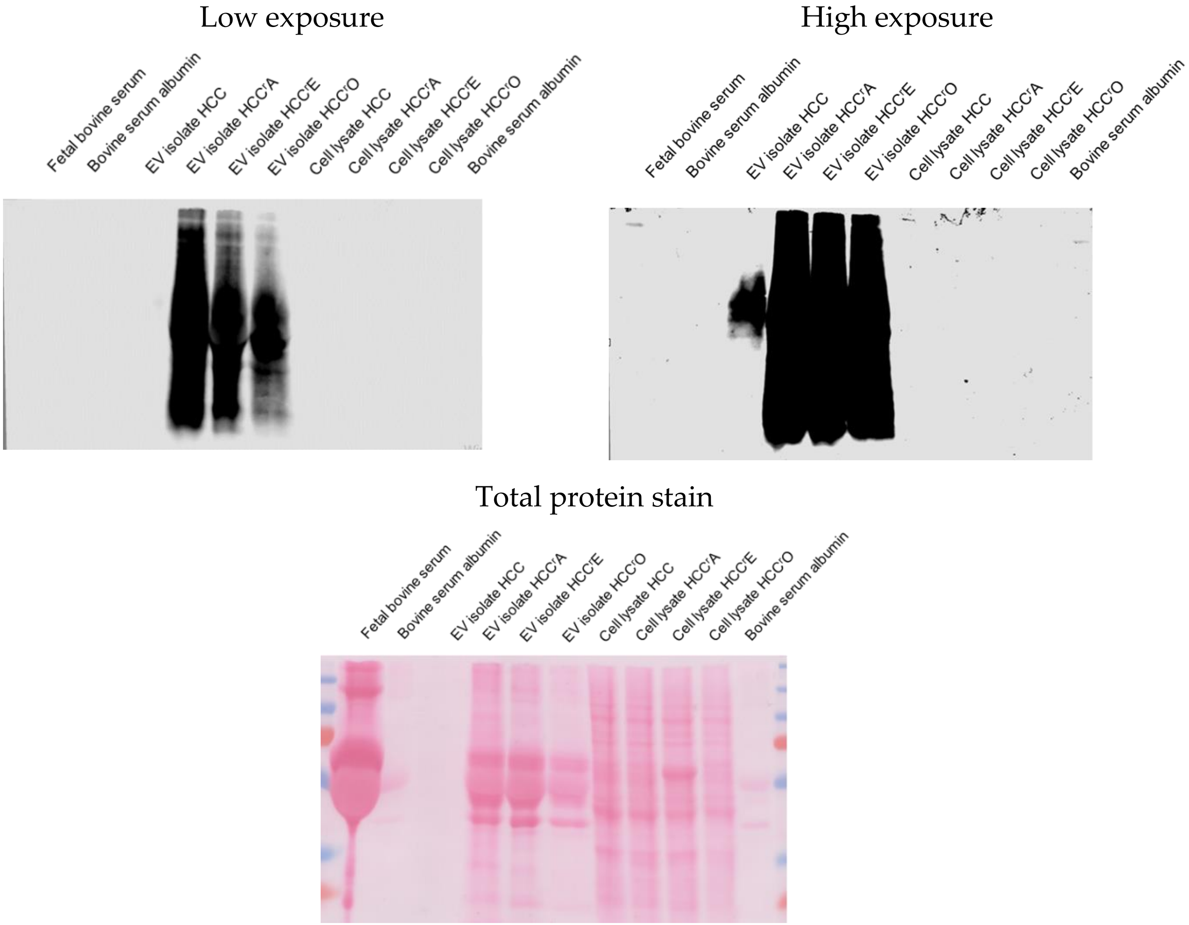

(c)

Supplementary Figure S2: Characterisation of extracellular vesicles (EVs): (a) EVs isolated from 16HBE14o-, HCC4006 and EGFR-TKI-resistant HCC4006 cells were analysed for size and size distribution using dynamic light scattering. (b) Size and round shaped morphology of isolated EVs was confirmed by scanning electron microscopy. (c) EVs, FBS, BSA and cell extracts were analysed for protein expression of exosome-enriched marker CD63 in HCC, HCC^{r}E and HCC^{r}O cells. 10 µL of FBS, EV isolate, 5 µg of BSA and 30 µg of cell extract were blotted on a nitrocellulose membrane and stained with Ponceau S total protein stain or a CD63-specific antibody (n = 1).

Supplementary Table S1: List of antibodies and dilutions

**Antibody**	**Dilution Western blotting**	**Dilution Immunocytochemistry**
Axl (C89E7) Rabbit mAb #8861	1:1000	-
E-cadherin (24E10) Rabbit mAb #3195	1:1000	1:1600
N-cadherin (D4R1H) XP® Rabbit mAb # 13116	1:1000	1:100
Vimentin (D21H3) XP® Rabbit mAb #5741	1:1000	-
ZEB1 (E2G9Y) XP® Rabbit mAb #70512	1:1000	-
CD63 Monoclonal Antibody (Ts63) #10628D	1:250	
IRDye® 800CW Goat anti-Rabbit IgG Secondary Antibody #926-32211	1:10,000	
IRDye® 800CW Goat anti-Mouse IgG Secondary Antibody #926-32210	1:10,000	
IRDye® 680LT Goat anti-Mouse IgG Secondary Antibody #926-68020	1:20,000	
IRDye® 680LT Goat anti-Rabbit IgG Secondary Antibody #926-68021	1:20,000	

Supplementary Table S2: List of doubling times and IC_{50} values of the parental and resistant HCC4006 cell lines

Cell line	Doubling time [h]	Inhibitor	IC_{50} [nM]
HCC4006	41.8 ± 7.5	Afatinib	3.601 ± 0.81
		Erlotinib	53.09 ± 2.34
		Gefitinib	26.23 ± 15.87
		Osimertinib	3.75 ± 2.02
HCC4006^{r}AFA$^{0.1}$	53.7 ± 16.7	Afatinib	>100
		Osimertinib	>1000
HCC4006^{r}ERLO$^{0.5}$	54.0 ± 5.5	Erlotinib	>1000
		Osimertinib	>1000
HCC4006^{r}GEFI$^{0.5}$	61.0 ± 12.4	Gefitinib	>1000
		Osimertinib	>1000
HCC4006^{r}OSI$^{0.5}$	66.7 ± 12.9	Afatinib	579.6
		Erlotinib	>1000
		Gefitinib	>1000
		Osimertinib	>1000

Supplementary Table S3: Primer sequences and efficiencies

**Gen/miRNA/ snRNA**	**Forward Primer**	**Reverse Primer**	**Efficiency [%]**
ALDH1A1	CGGGAAAAGCAATCTGAAGAGG	GATGCGGCTATACAACACTGGC	90.78
ACTB	CACCATTGGCAATGAGCGGTTC	AGGTCTTTGCGGATGTCCACGT	96.81
CD24	CACGCAGATTTATTCCAGTGAAA	GACCACGAAGAGACTGGCTGTT	90.13

CD44	CCAGAAGGAACAGTGGTTTGGC	ACTGTCCTCTGGGCTTGGTGTT	90.58
CDH1	GCCTCCTGAAAAGAGAGTGGAAG	TGGCAGTGTCTCTCCAAATCCG	98.83
CDH2	CCTCCAGAGTTTACTGCCATGAC	GTAGGATCTCCGCCACTGATTC	90.53
GAPDH	GTCTCCTCTGACTTCAACAGCG	ACCACCCTGTTGCTGTAGCCAA	95.35
MMP2	AGCGAGTGGATGCCGCCTTTAA	CATTCCAGGCATCTGCGATGAG	97.68
PTEN	TGAGTTCCCTCAGCCGTTACCT	GAGGTTTCCTCTGGTCCTGGTA	94.88
RNU6	GTCGTGAAGCGTTCCA	CAGGTCCAGTTTTTTTTTTTTTTTAAA	98.65
SNAI1	TGCCCTCAAGATGCACATCCGA	GGGACAGGAGAAGGGCTTCTC	91.68
SNAI2	ATCTGCGGCAAGGCGTTTTCCA	GAGCCCTCAGATTTGACCTGTC	92.56
TWIST1	GCCAGGTACATCGACTTCCTCT	TCCATCCTCCAGACCGAGAAGG	95.42
VIM	AGGCAAAGCAGGAGTCCACTGA	ATCTGGCGTTCCAGGGACTCAT	91.15
ZEB1	GGCATACACCTACTCAACTACGG	TGGGCGGTGTAGAATCAGAGTC	100.32
ZEB2	AATGCACAGAGTGTGGCAAGGC	CTGCTGATGTGCGAACTGTAGG	96.92
miR-9-5p	GCAGTCTTTGGTTATCTAGCTGT	GTCCAGTTTTTTTTTTTTTTTCATACA	86.724
miR-21-5p	TCAGTAGCTTATCAGACTGATG	CGTCCAGTTTTTTTTTTTTTTTCAAC	113.029
miR-34a-5p	GGCAGTGTCTTAGCTGGT	TCCAGTTTTTTTTTTTTTTTACAACCA	106.256
miR-99a-5p	CAGAACCCGTAGATCCGA	GTCCAGTTTTTTTTTTTTTTTCACAAG	101.836
miR-126-3p	GCAGTCGTACCGTGAGTAA	CCAGTTTTTTTTTTTTTTTCGCAT	109.004
miR-139-5p	GTCTACAGTGCACGTGTC	CCAGTTTTTTTTTTTTTTTACTGGAG	109.196
miR-155-5p	CGCAGTTAATGCTAATCGTGATA	GGTCCAGTTTTTTTTTTTTTTTAACC	102.858
miR-183-5p	GCAGTATGGCACTGGTAGA	GGTCCAGTTTTTTTTTTTTTTTAGTGA	108.645
miR-192-5p	CAGCTGACCTATGAATTGACA	TCCAGTTTTTTTTTTTTTTTGGCT	104.902
miR-200c-3p	AGTAATACTGCCGGGTAATGA	TCCAGTTTTTTTTTTTTTTTCCATCA	99.441
miR-203a-3p	AGGTGAAATGTTTAGGACCAC	GTCCAGTTTTTTTTTTTTTTTCTAGTG	103.881
miR-205-5p	CCTTCATTCCACCGGAGT	GGTCCAGTTTTTTTTTTTTTTTCAGA	114.939
miR-374a-5p	GCGCAGTTATAATACAACCTGA	GGTCCAGTTTTTTTTTTTTTTTCACT	98.679
miR-378a-5p	TCCTGACTCCAGGTCCT	GGTCCAGTTTTTTTTTTTTTTTACACA	106.167
miR-451a	CGCAGAAACCGTTACCA	GTCCAGTTTTTTTTTTTTTTTAACTCA	101.153
miR-544a	GCAGATTCTGCATTTTTAGCAAG	GGTCCAGTTTTTTTTTTTTTTTGAAC	83.946
Universal RT Primer	CAGGTCCAGTTTTTTTTTTTTTTTVN (V = A, C and G; N = A, C, G and T)		

Supplementary Table S4: Mimic and negative control mimic sequence

Mimics	Sense (5′-3′)	Antisense (5′-3′)
miR-205-5p	UCCUUCAUUCCACCGGAGUCUC	GACUCCGGUGGAAUGAAGGAUU
miR-205-5p NC	UUCUCCGAACGUGUCACGUTT	ACGUGACACGUUCGGAGAATT

Supplementary Table S5: Size of extracellular vesicles determined by dynamic light scattering

Cell line	Peak Size [nm]	Z-Average [nm]	Polydispersity Index
16HBE14o-	125.4	189.6	0.240
HCC4006	120.5	185.5	0.399
HCC4006^{r}AFA$^{0.1}$	103.6	195.9	0.317
HCC4006^{r}ERLO$^{0.5}$	58.3	121.3	0.343
HCC4006^{r}GEFI$^{0.5}$	101.8	151.2	0.289
HCC4006^{r}OSI$^{0.5}$	106.4	194.4	0.367

Supplementary Table S6: Size of extracellular vesicles from HCC^{r}A determined by scanning electron microscopy

HCC4006^{r}AFA$^{0.1}$	**Size [nm]**	**SD [nm]**
	113.8	21.6
