# Supplementary material for: Loss of Key EMT-Regulating miRNAs Highlight the Role of ZEB1 in EGFR Tyrosine Kinase Inhibitor-Resistant NSCLC
Source: Int J Mol Sci. 2023 Sep 29;24(19):14742. doi: 10.3390/ijms241914742 (PMC10573279; doi:10.3390/ijms241914742)

Western Blot Original Files

Protein Expression of EMT Markers (Figure 2e)

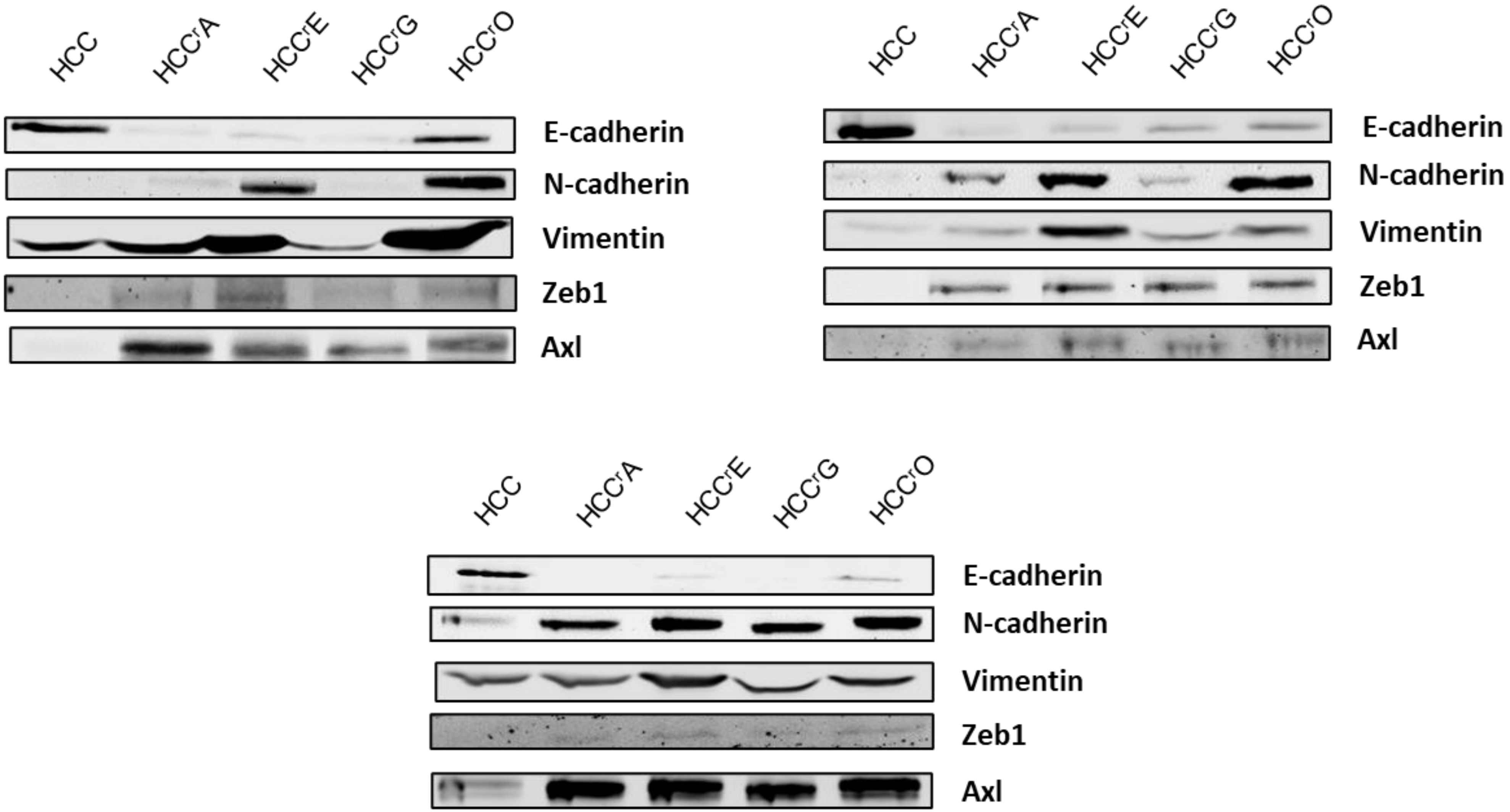

Biological Replicate 1

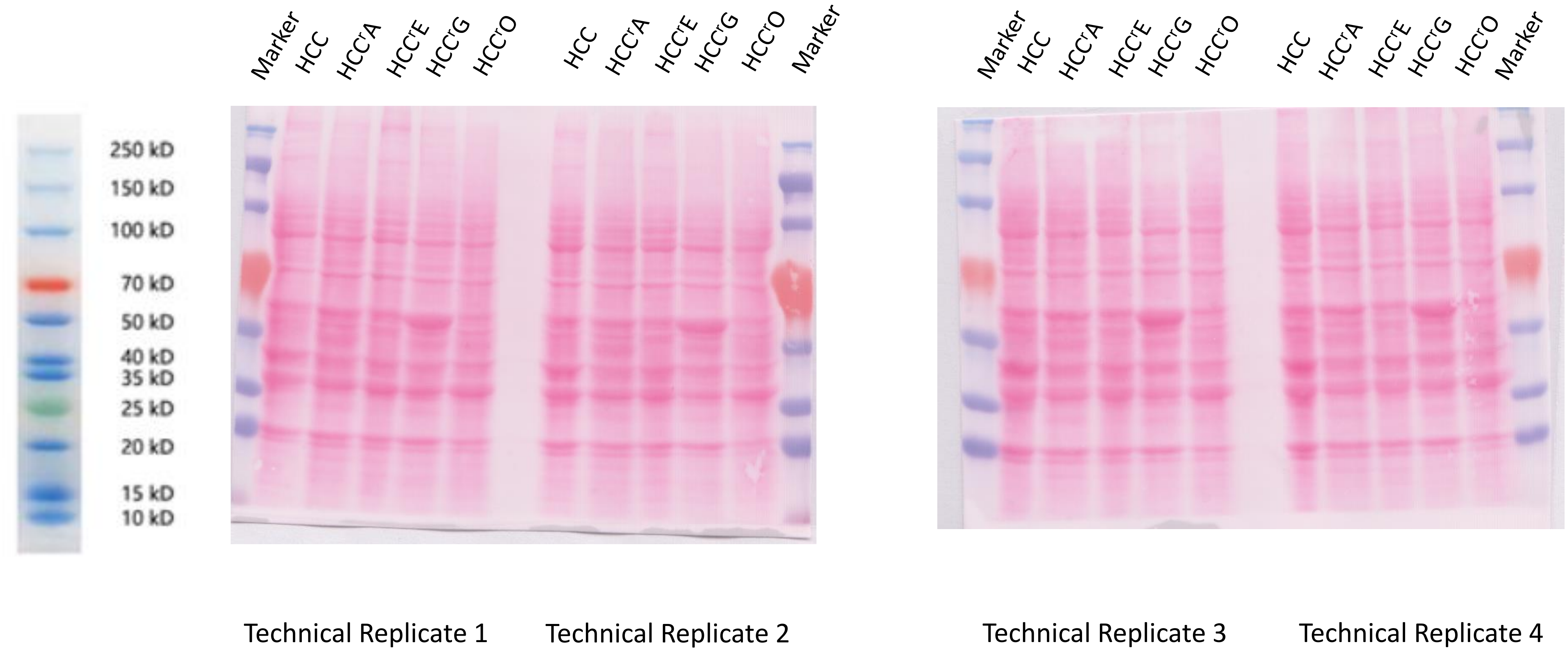

Biological Replicate 1

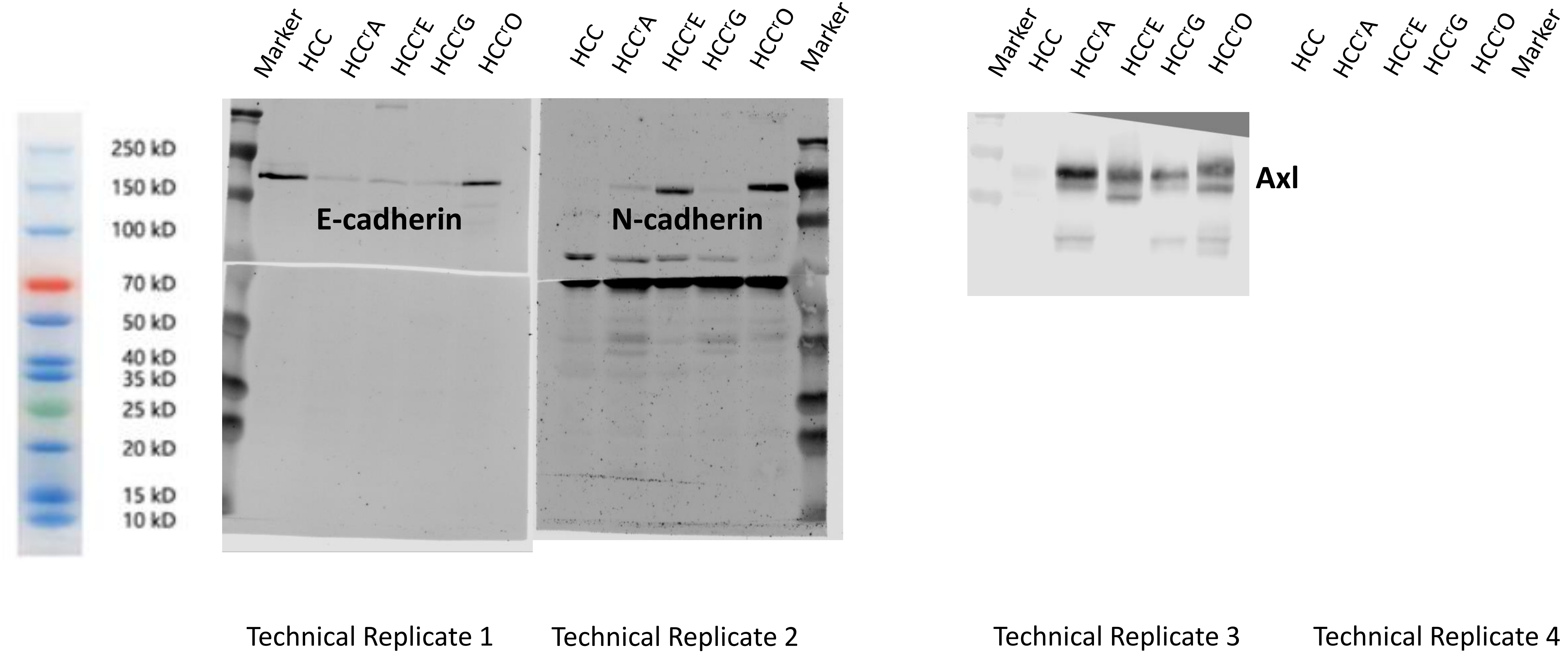

Biological Replicate 1

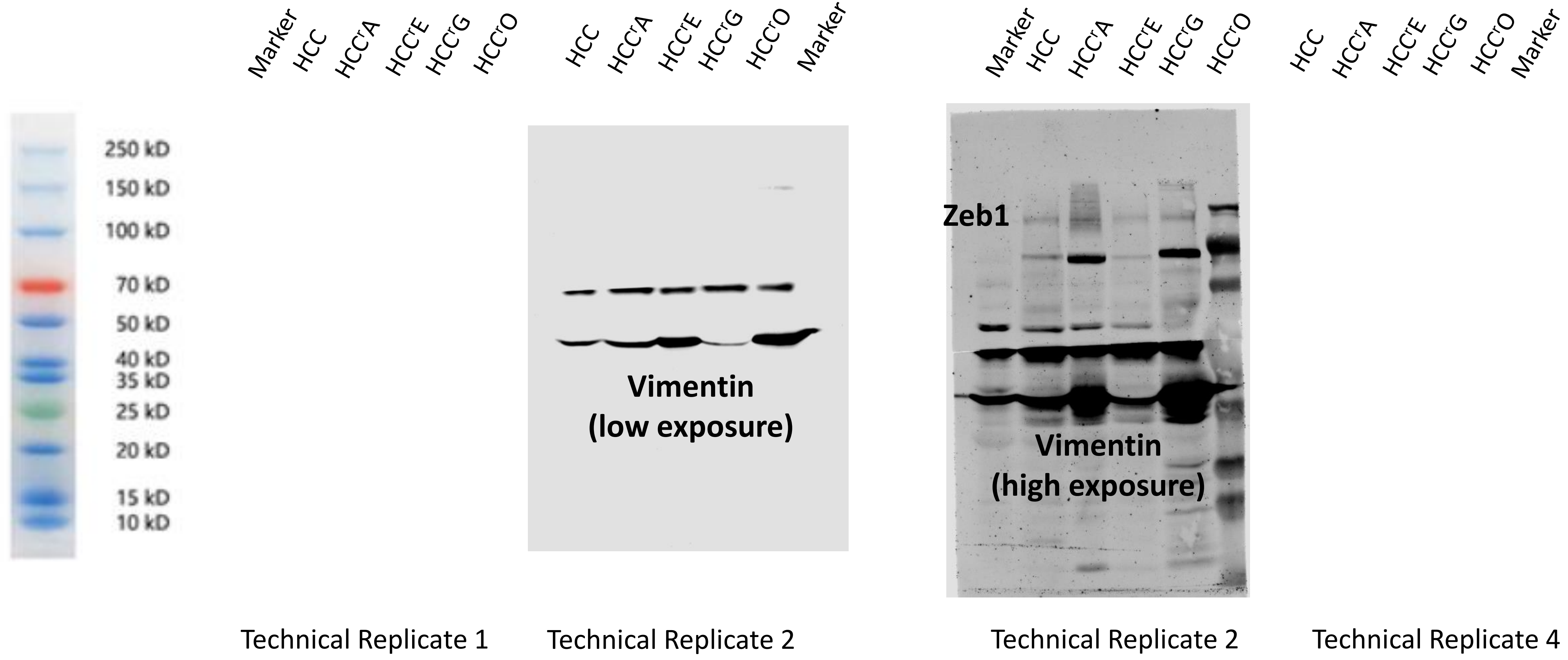

Biological Replicate 2

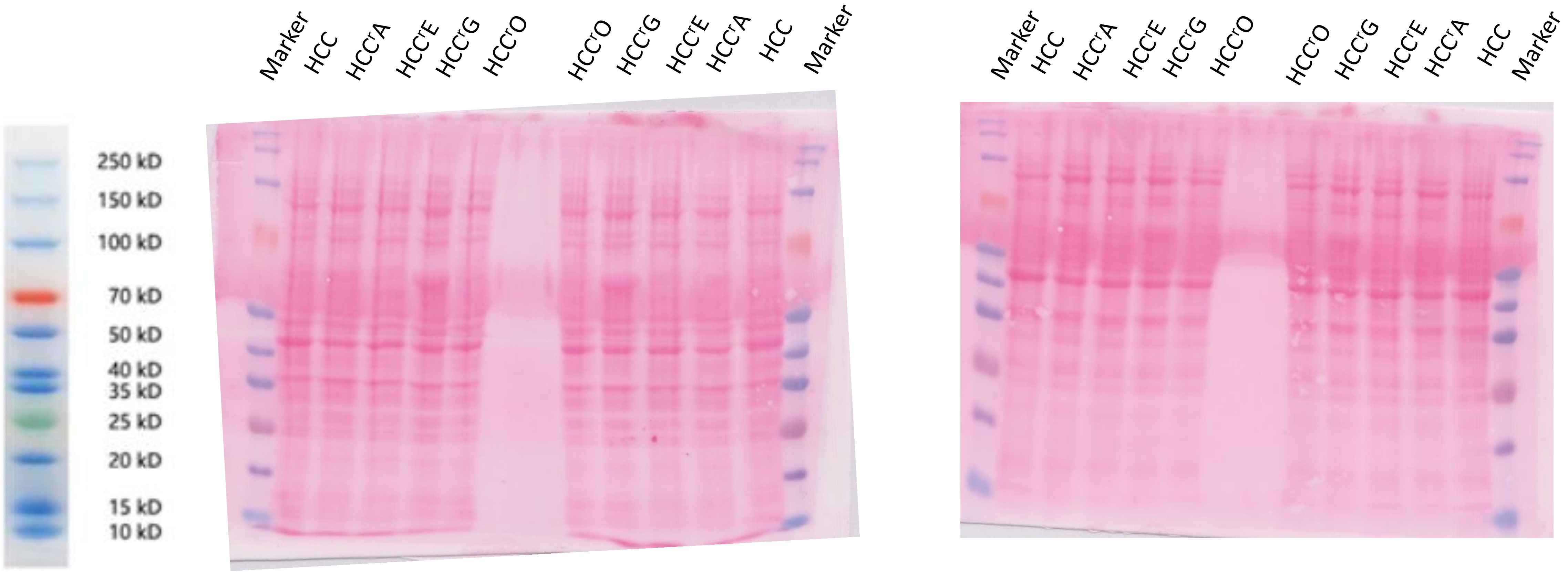

Technical Replicate 1 Technical Replicate 2 Technical Replicate 3 Technical Replicate 4

Biological Replicate 2

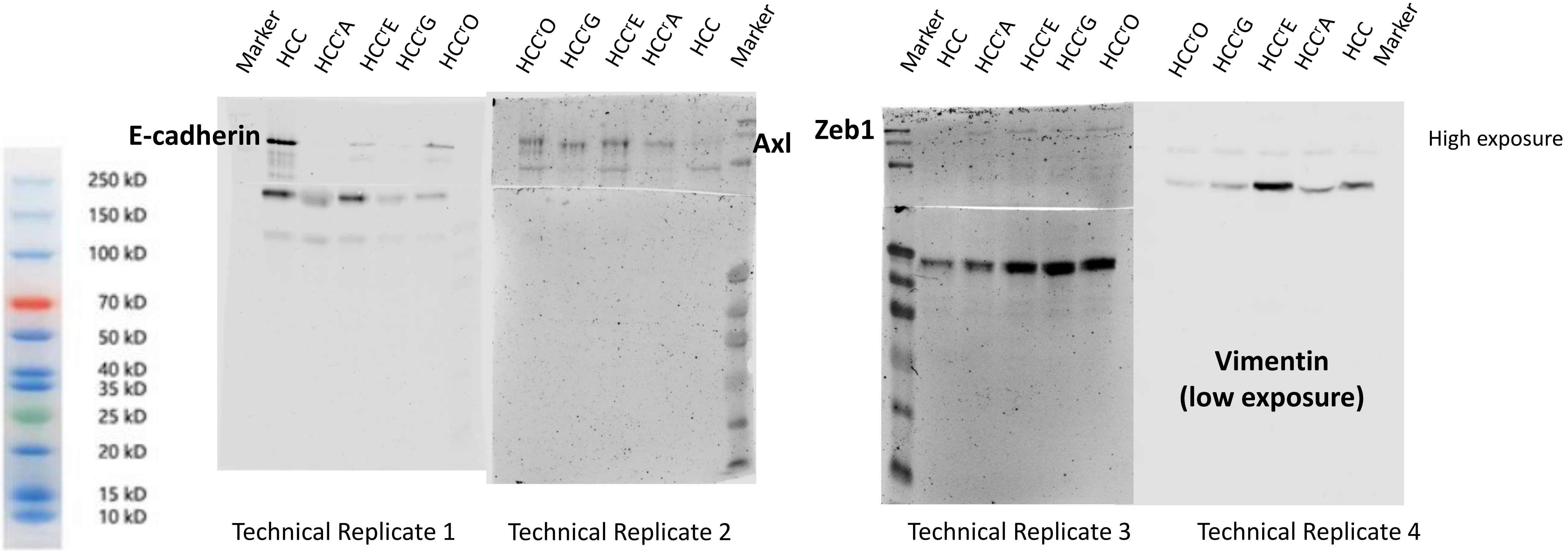

Biological Replicate 2

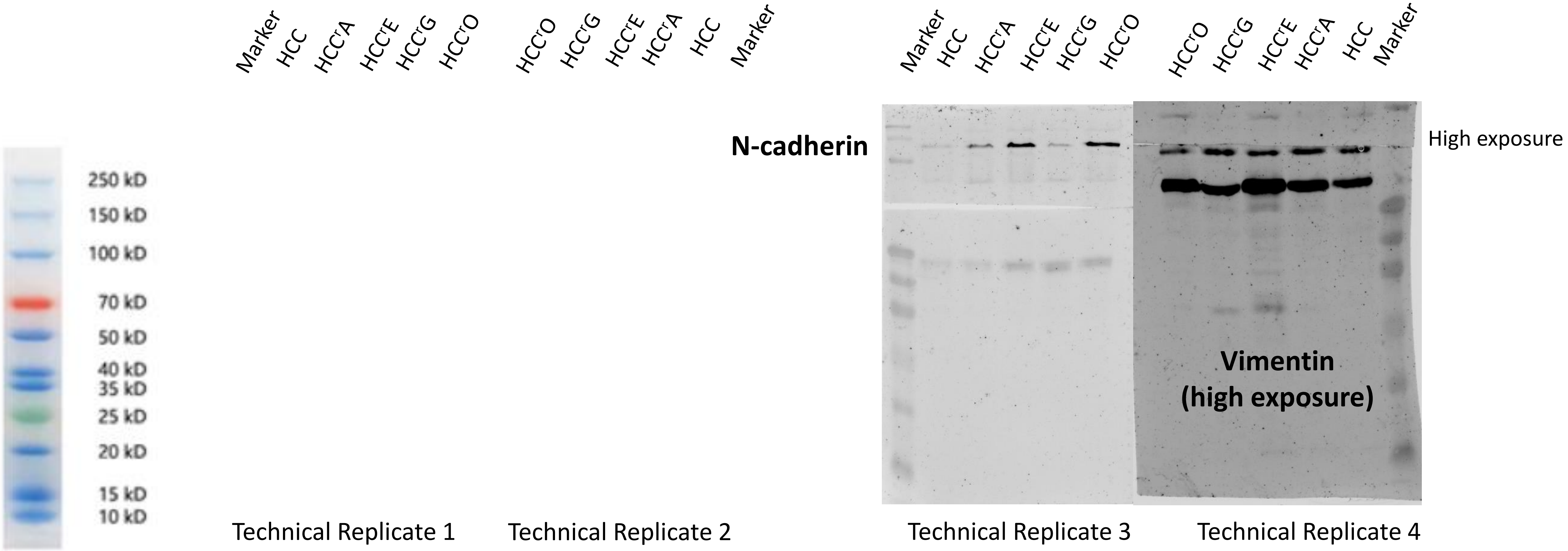

Biological Replicate 3

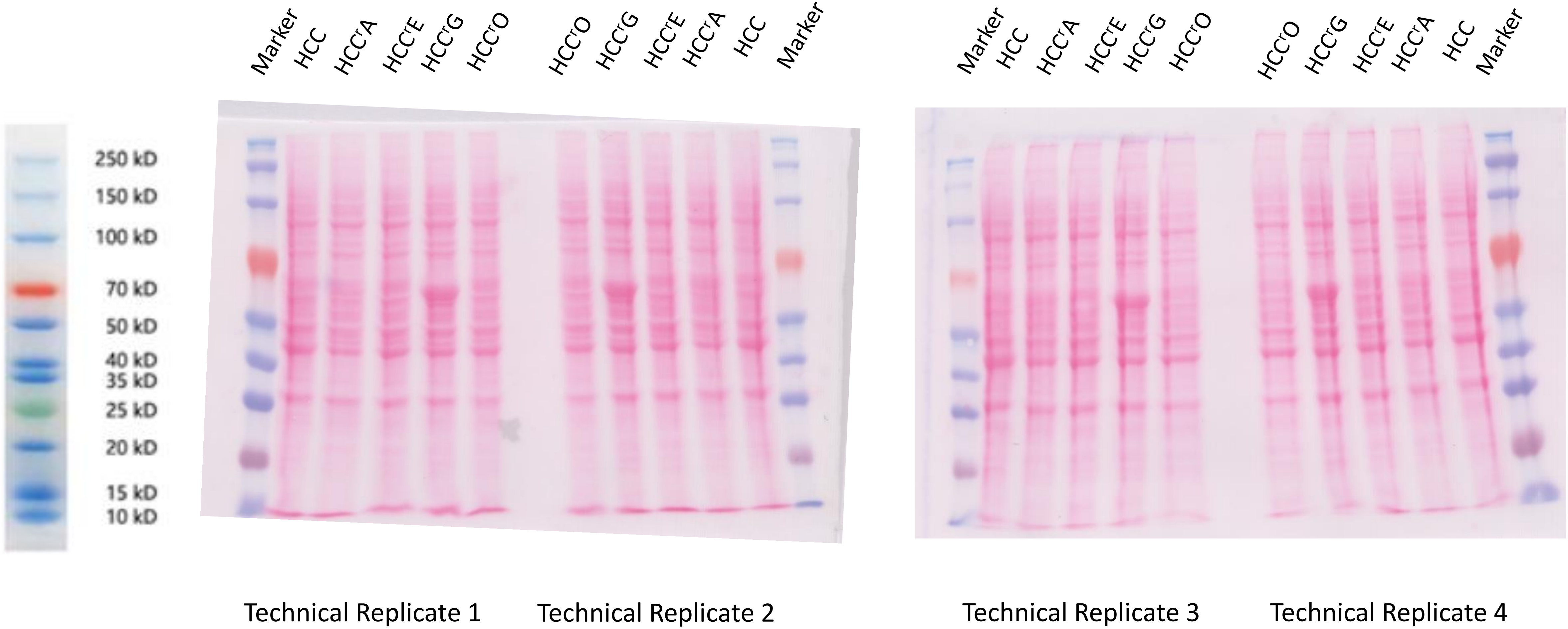

Biological Replicate 3

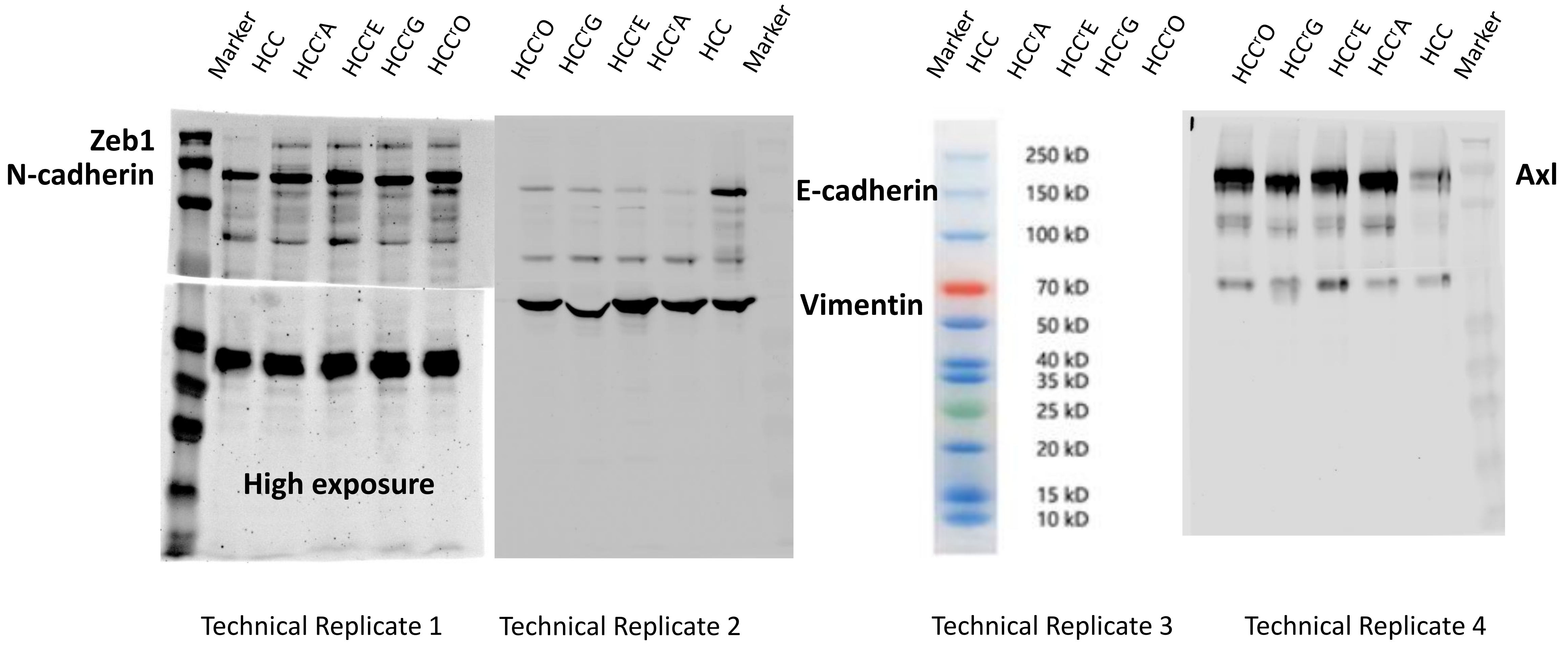

Biological Replicate 3

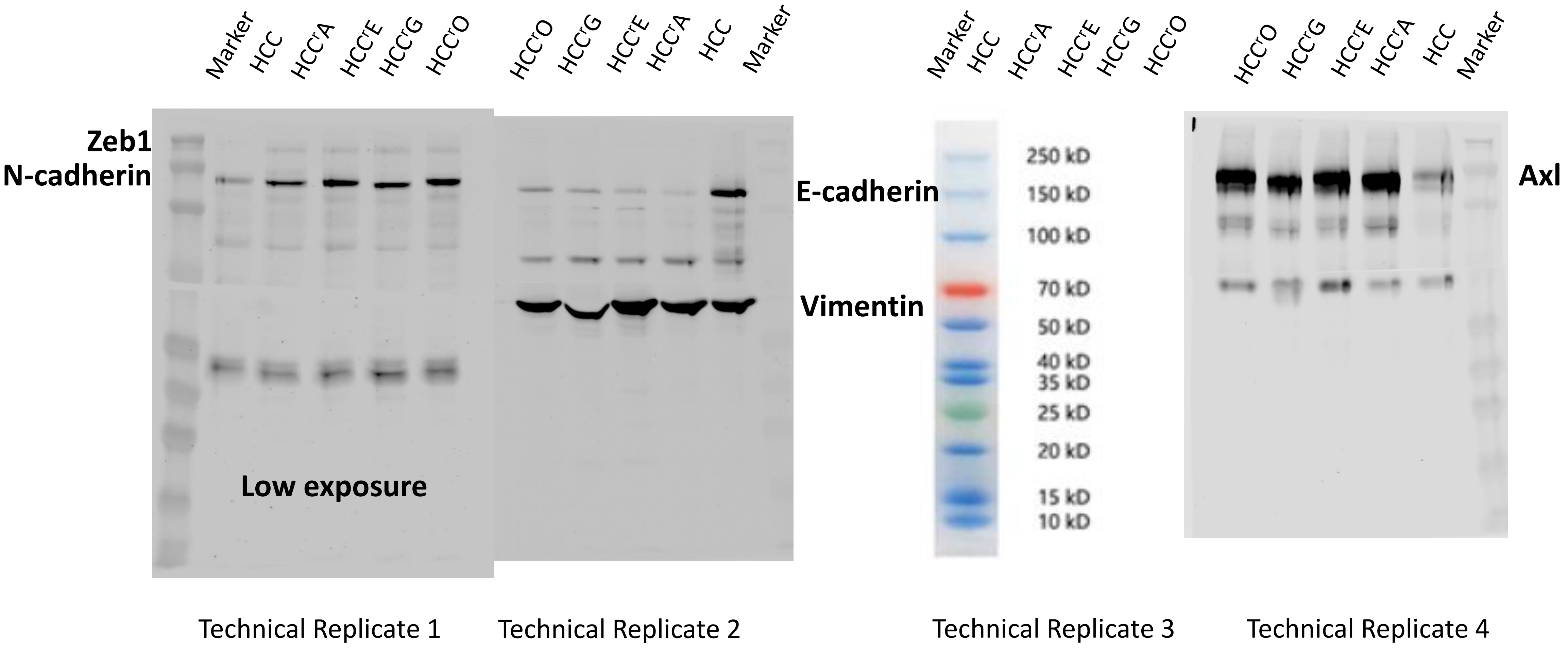

Supplement: Supplementary file 1 [file ijms-24-14742-s001.zip › Western Blot Original Files.pdf]
